# Microstructural variation of hippocampal substructures across childhood and adolescence quantified with high-gradient diffusion MRI

**DOI:** 10.1038/s42003-026-09622-x

**Published:** 2026-02-12

**Authors:** Bradley G. Karat, Sila Genc, Erika P. Raven, Marco Palombo, Ali R. Khan, Derek K. Jones

**Affiliations:** 1 Robarts Research Institute, Western University, London, ON Canada; 2 Centre for Functional and Metabolic Mapping, Western University, London, ON Canada; 3 Department of Neurosurgery, The Royal Children’s Hospital, Melbourne, VIC Australia; 4 Cardiff University Brain Research Imaging Centre (CUBRIC), Cardiff University, Cardiff, UK; 5 Center for Biomedical Imaging, Department of Radiology, New York University Grossman School of Medicine, New York, NY USA; 6 School of Computer Science and Informatics, Cardiff University, Cardiff, UK; 7 Department of Medical Biophysics, Western University, London, ON Canada

**Keywords:** Neuronal development, Hippocampus

## Abstract

The hippocampus plays a crucial role in cognition, yet its microstructural development during childhood and adolescence remains poorly understood. Here, we investigate age-related differences in hippocampal microstructure using diffusion MRI with ultra-strong gradients (300 mT/m) in a cohort of 88 participants aged 8–19 years. Surface-based hippocampal modelling was combined with established microstructural approaches, and a more advanced biophysical model (Soma and Neurite Density Imaging: SANDI) suited for studying cortical microstructure. Hippocampal volume, gyrification, and thickness remained stable across this developmental window, however we observed significant differences across age related to MR-derived neurite and soma parameters. Diffusion-derived changes across age were found to be correlated with adult microstructure maps related to myelin and iron content, synaptic density, and hippocampal interneurons derived from MRI, PET and histology. These findings highlight age-related differences of MR-derived neurite and soma parameters in the human hippocampus during late childhood and adolescence, offering insights into structural maturation during this critical period.

## Abstract (web summary)

Neurite and soma modelling with high-gradient in vivo diffusion MRI revealed distinct age-related profiles of hippocampal substructures in late childhood and adolescence.

## Introduction

The hippocampus is a widely studied yet enigmatic archicortical region that is typically parcellated into mesoscopic subfields, which differ in both structure and function^1,2^. Part of its mystery arises from its relatively uncharacterized development. One hypothesis posits that the evolutionary development of the neocortex arose from a primordial hippocampus and amygdala, highlighting its importance in acquiring higher-order cognitive functions^3–5^. However, little is known about how the hippocampus develops on the timescale of a human lifespan, particularly in late childhood (~6–12 years) and adolescence (~12–18 years). This characterization is critical to better understand the formation of human cognition, and the principal role the hippocampus has in functions like episodic and semantic memory, spatial navigation, emotion, behavior, and more^6–9^.

While important, it is a challenging task to study the hippocampus during the early years of human development. Contemporary research has used noninvasive methods, such as magnetic resonance imaging (MRI) to examine the hippocampus and its subfields across these early developmental stages^10–21^. Most studies have focused on volumetric changes, where it has generally been shown that hippocampal volume increases across childhood and adolescence, likely capturing an expansion of cognitive capacity^13–16,18,21^. However, there have been conflicting results, with studies finding variable patterns of age-related hippocampal volume changes^11,15,18,21^. While volume does appear to be sensitive to developmental changes, it is a coarse measure, which is unspecific towards the intrahippocampal gray matter (GM). This includes components such as glial cells, neurites, soma and other micron-scale structures (collectively termed microstructure) that are responsible for the computations which engender hippocampal function and are of critical importance in both health and disease.

The development of GM microstructure is generally characterized by rapid growth of dendrites, axons, and synaptogenesis in early adolescence, which are pruned during later adolescent years, as shown in both human^22,23^ and nonhuman primates^24^. Diffusion MRI (dMRI) is a technique, which sensitizes the MRI signal to the micron-scale movement of water, which can be leveraged to study developmental patterns of microstructure^25,26^. Previous studies using diffusion tensor imaging (DTI)^27^ to capture hippocampal microstructural development have generally found a negative correlation between mean diffusivity (MD) and age in early childhood, while age-related changes in fractional anisotropy (FA) have been more variable^10,14,17,20^. However, these findings are not specific to any particular microstructural property and could be a result of changes to axon or dendrite density, myelination, soma-related changes, or other micron-level alterations^28–30^. Recent advances in MRI hardware, including stronger gradients^31–33^ and new modeling approaches^34^ appear promising to disentangle apparent soma and neurite contributions to the dMRI signal in vivo.

In this work, we examined age-related differences in hippocampal microstructure using dMRI data acquired using an ultra-strong gradient (300 mT/m) MRI scanner in a sample of children and adolescents (aged 8–19 years)^35,36^. Using HippUnfold, a hippocampal surface-based approach, we investigated age and sex-related differences in macro- and microstructure across the subfields and long-axis^37^. In particular, the soma and neurite density imaging (SANDI)^34^ model was used to derive measures related to both the soma and neurite. The neurite orientation dispersion and density imaging model (NODDI)^38^ and DTI^27^ were also used to compare microstructure differences across age. Utilizing the salient orientation information from dMRI, we also determined if there were shifts in diffusion orientation across age, which may be related to the development of the complex but organized intrahippocampal circuitry. Finally, we derived surface maps, which capture age effects for all macro- and microstructural measures. We then correlated these maps with metrics derived from histology of adult (post-mortem) human brains, PET, and high-resolution MRI from previous research to postulate what the age-related differences might be capturing in terms of known microstructure. Overall, we report distinct neurite and soma developmental profiles in the human hippocampus during late childhood/adolescence for the first time.

## Results

### Age and sex-related differences in subfield volume and macrostructure

**Fig. 1:**
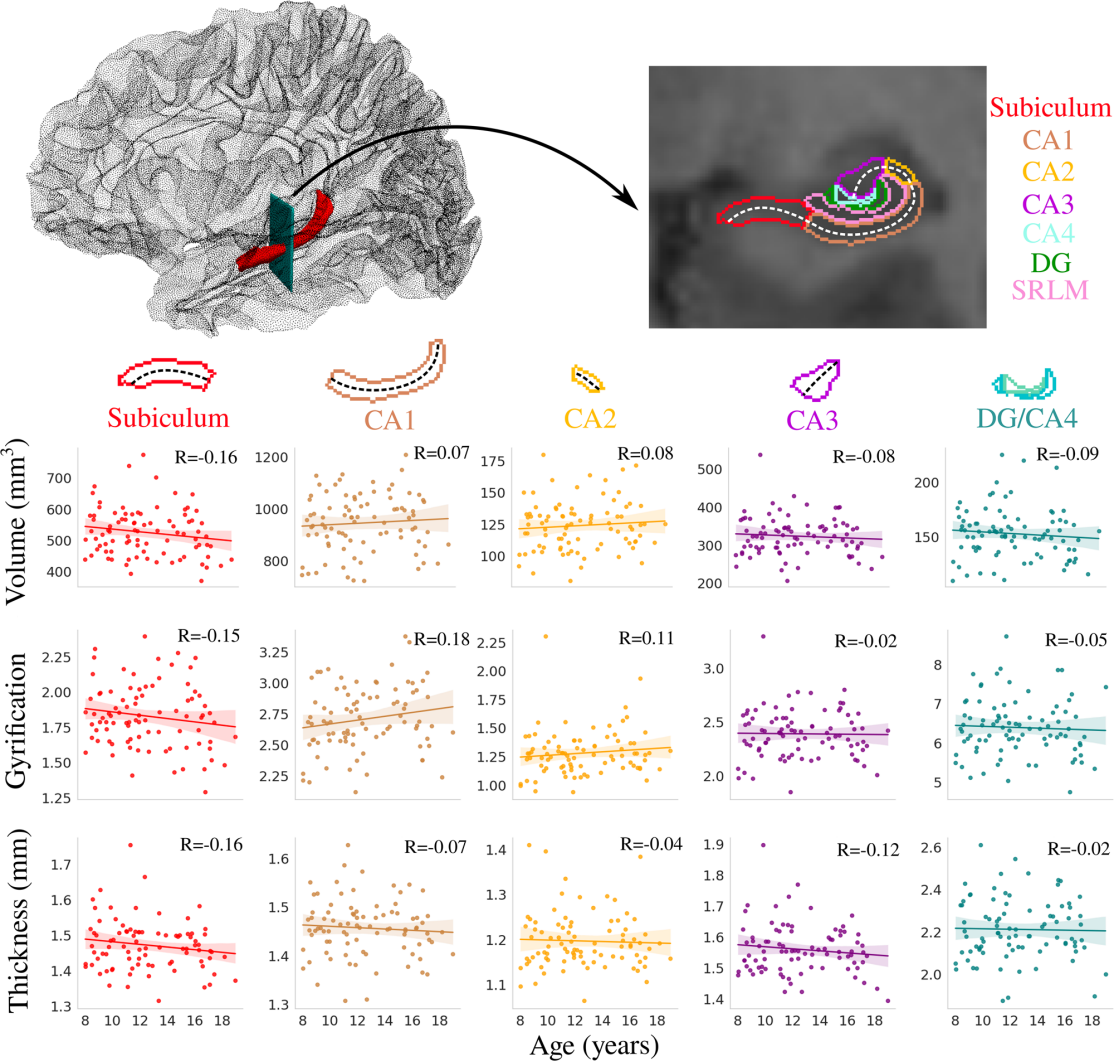
Correlation between age and subfield-averaged macrostructural measures of volume, gyrification, and thickness. The top left figure depicts the 3D location of the hippocampus (shown in red), and the arrow represents the position of the coronal slice shown on the top right figure. Colors represent hippocampal subfields, and relationships are quantified using Pearson’s correlation coefficient (*R*). The dashed lines approximately represent the midthickness surface, which gyrification and thickness were calculated and then averaged on (note that the surface excludes the SRLM). As well, the DG and CA4 were averaged together. Translucent bands around the regression line show the 95% confidence interval based on 1000 bootstrap resamples of the data. CA cornu ammonis, DG dentate gyrus, SRLM stratum radiatum lacunosum moleculare.

Previous research has typically analyzed the volume of the hippocampus across development^13–15,19^. Figure 1 depicts the correlation between age and subfield-averaged macrostructural measures of volume, gyrification, and thickness. No significant interaction was found between age and hemisphere for volume (*F*(1864) = 0.001, *p* = 0.974), gyrification (*F*(1864) = 0.104, *p* = 0.747), or thickness (*F*(1864) = 0.554, *p* = 0.457), suggesting that the hemispheres display similar age-related differences in subfield macrostructure (Supplementary Fig. S1 and Supplementary Table S1). Thus, hemisphere data (i.e., between left and right hippocampus) was averaged within participants.

No significant correlations were found between age and any subfield-averaged macrostructural measure (Fig. 1). Similar results were found for anterior-posterior (AP) averaged macrostructure, except for posterior gyrification, which had a significant correlation with age (*R* = −0.27, *p* < 0.01) (Supplementary Fig. S2). As well, no significant correlation between age and subfield volume was found using FreeSurfer (Supplementary Fig. S3), corroborating the result seen in Fig. 1.

**Fig. 2:**
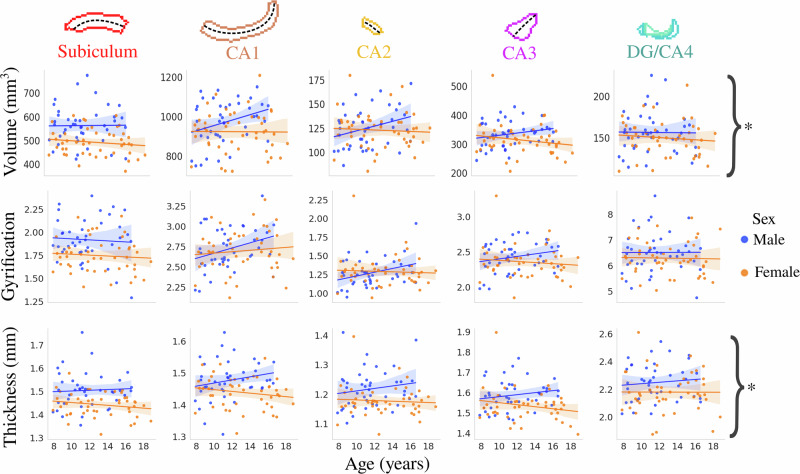
Relationship between age and macrostructure by hippocampal subfield and stratified by sex. Though the subfields are plotted separately here, the asterisks represent metrics with a significant interaction between age and sex (i.e., subfields are combined) after FDR correction (*p* < 0.05). Translucent bands around the regression line show the 95% confidence interval based on 1000 bootstrap resamples of the data.

Figure 2 depicts the correlation between age and subfield-averaged macrostructural measures of volume, gyrification, and thickness stratified by sex. Interestingly, it appears that males generally have positive correlations between age and subfield volume, gyrification, and thickness, while females showed little correlation with age. The interaction between age and sex (combined subfields) was significant after false-discovery rate (FDR) correction for volume (*F*(1424) = 6.03, *p*-adjusted = 0.040) and thickness (*F*(1424) = 4.966, *p*-adjusted = 0.040), while not significant for gyrification (*F*(1424) = 1.573, *p*-adjusted = 0.211) (Supplementary Table S2).

### Age and sex-related differences in subfield microstructure

Supplementary Fig. S4 depicts the relationship between age and subfield-averaged partial volume maps of CSF, GM, and WM. No significant correlations were found between age and the CSF partial volume measure. In CA1, CA2, CA3, and the DG/CA4, the GM tissue probability is generally between 0.8 and 1, while the CSF probability is between 0 and 0.07, suggesting that the microstructure measures sampled on the midthickness surface are mostly within the GM. The subiculum has a higher WM tissue probability (0.2–0.4), which is expected given the presence of the highly myelinated perforant path.

**Fig. 3:**
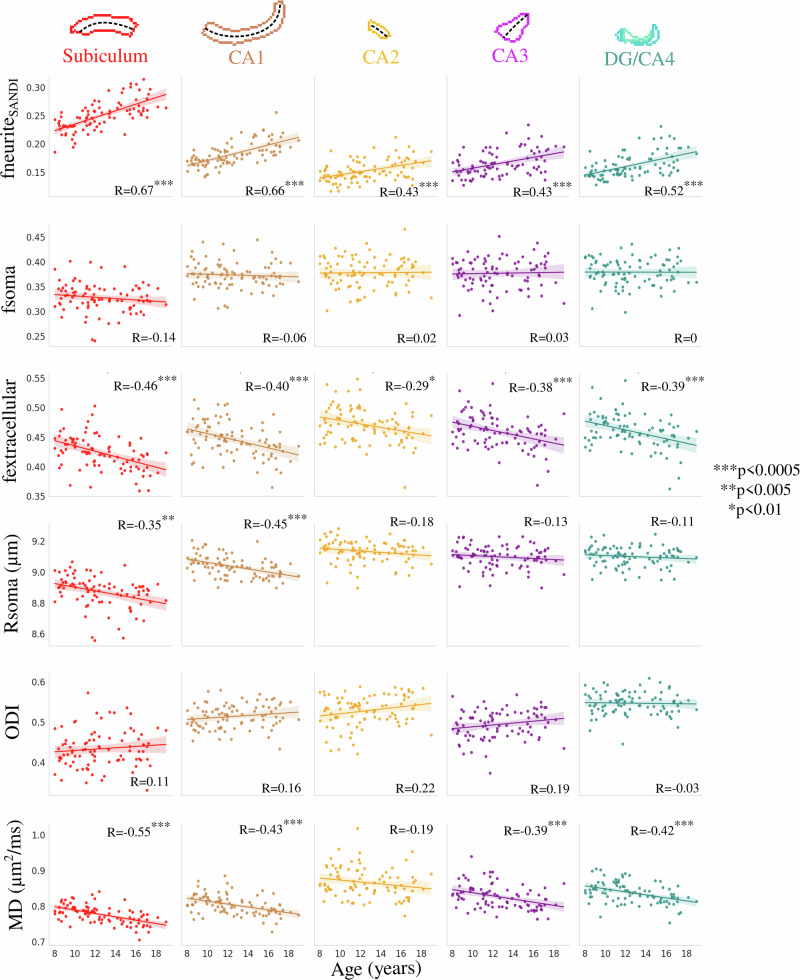
Correlation between age and subfield-averaged microstructural measures. Microstructural measures include neurite (fneurite_SANDI_), soma (fsoma), and extracellular (fextracellular) MR signal fractions, soma radius (Rsoma), orientation dispersion index (ODI) and mean diffusivity (MD). Colors represent hippocampal subfields, and relationships are quantified using Pearson’s correlation coefficient (*R*) with the significance represented by asterisk(s), which is the probability that two uncorrelated variables could produce a correlation similar to the observed correlation value. The dashed lines approximately represent the midthickness surface, which the metrics were sampled and then averaged on. Translucent bands around the regression line show the 95% confidence interval based on 1000 bootstrap resamples of the data. CA cornu ammonis, DG dentate gyrus.

Figure 3 depicts the correlation between subfield-averaged microstructural measures and age using SANDI (neurite, soma, and extracellular MR signal fractions as well as MR apparent soma radius), NODDI (orientation dispersion index), and DTI (MD) metrics. No significant interaction was found between age and hemisphere for fneurite_SANDI_ (*F*(1864) = 0.014, *p* = 0.906), fsoma (*F*(1864) = 0.001, *p* = 0.971), fextracellular (*F*(1864) = 0.014, *p* = 0.907), Rsoma (*F*(1864) = 0.576, *p* = 0.448), ODI (*F*(1864) = 0.089, *p* = 0.766), and MD (*F*(1864) = 1.223, *p* = 0.269), suggesting that the hemispheres display similar age-related differences in subfield microstructure (Supplementary Fig. S5 and Supplementary Table S1). Thus, hemisphere data were averaged within participants.

Many significant correlations were found between age and subfield-averaged microstructural measures (Fig. 3). In particular, fneurite_SANDI_ was significantly positively correlated with age across all subfields, while fextracellular and MD were significantly negatively correlated with age across all subfields (with the exception of CA2; Fig. 3). Rsoma was significantly negatively correlated with age only in the subiculum and CA1. ODI and fsoma were not significantly correlated with age in any subfield. Similar to fneurite_SANDI,_ fneurite_NODDI_ was significantly positively correlated with age across all subfields, while FA was not significantly correlated with age in any subfield (Supplementary Fig. S6). Interestingly, the age-related slopes seen in Fig. 3 were significantly different across the subfields for fneurite_SANDI_ (*F*(4424) = 4.354, *p*-adjusted = 0.011) and Rsoma (*F*(4424) = 3.086, *p*-adjusted = 0.047), suggesting that the subfields may have unique patterns of neurite and soma development (Supplementary Table S3). However, the slopes across age were not significantly different across the subfields for fsoma (*F*(4424) = 0.511, *p*-adjusted = 0.894), fextracellular (*F*(4424) = 0.274, *p*-adjusted = 0.894), ODI (*F*(4424) = 0.838, *p*-adjusted = 0.894), and MD (*F*(4424) = 0.281, *p*-adjusted = 0.894) (Supplementary Table S3).

**Fig. 4:**
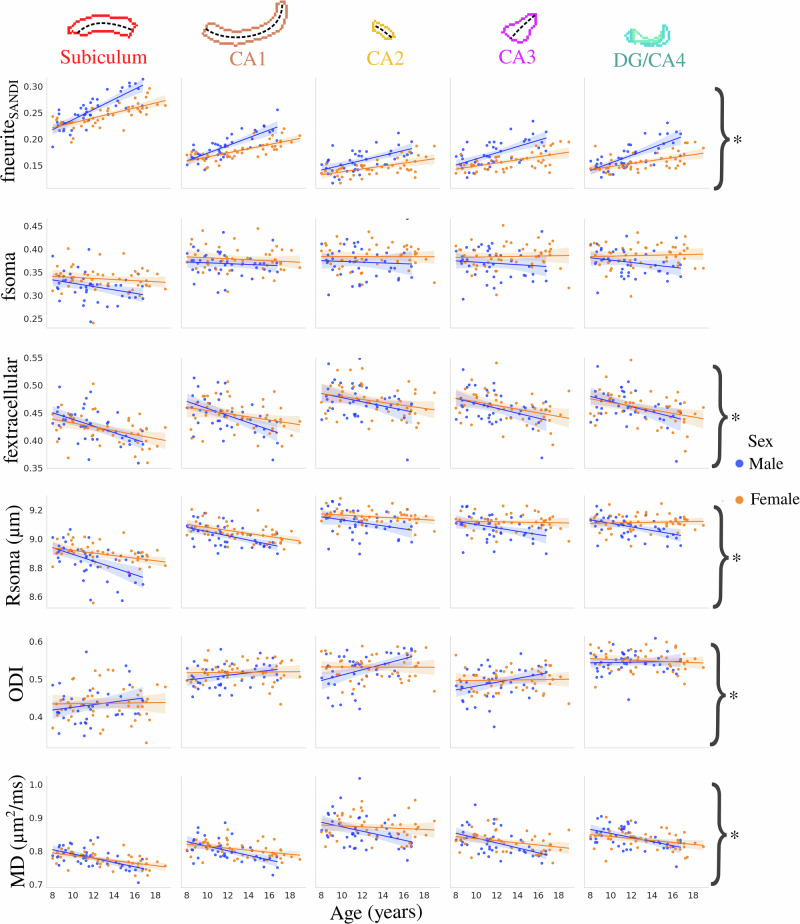
Relationship between age and microstructure by hippocampal subfield and stratified by sex. Though the subfields are plotted separately here, the asterisks represent metrics with a significant interaction between age and sex (i.e., subfields are combined) after FDR correction (*p* < 0.05). Translucent bands around the regression line show the 95% confidence interval based on 1000 bootstrap resamples of the data.

Figure 4 depicts the correlation between subfield-averaged microstructural measures and age stratified by sex. The interaction between age and sex was significant after FDR correction for fneurite_SANDI_ (*F*(1424) = 43.39, *p*-adjusted = 8 × 10^−10^), fextracellular (*F*(1424) = 4.509, *p*-adjusted = 0.04), Rsoma (*F*(1424) = 16.819, *p*-adjusted = 1.47 × 10^−04^), ODI (F(1424) = 10.392, *p*-adjusted = 0.002), and MD (*F*(1424) = 14.247, *p*-adjusted = 3.66 × 10^−04^) (Supplementary Table S2). The age-by-sex interaction was not significant for fsoma (*F*(1424) = 2.673, *p*-adjusted = 0.103). The correlation between subfield-averaged fneurite_NODDI_ and FA across age stratified by sex is shown in Supplementary Fig. S7. The interaction between age and sex was significant after FDR correction for fneurite_NODDI_ (*F*(1424) = 32.02, *p*-adjusted = 5 × 10^−8^) while not significant for FA (*F*(1424) = 0.32, *p*-adjusted = 0.57).

### Age-related differences in long-axis microstructure

**Fig. 5:**
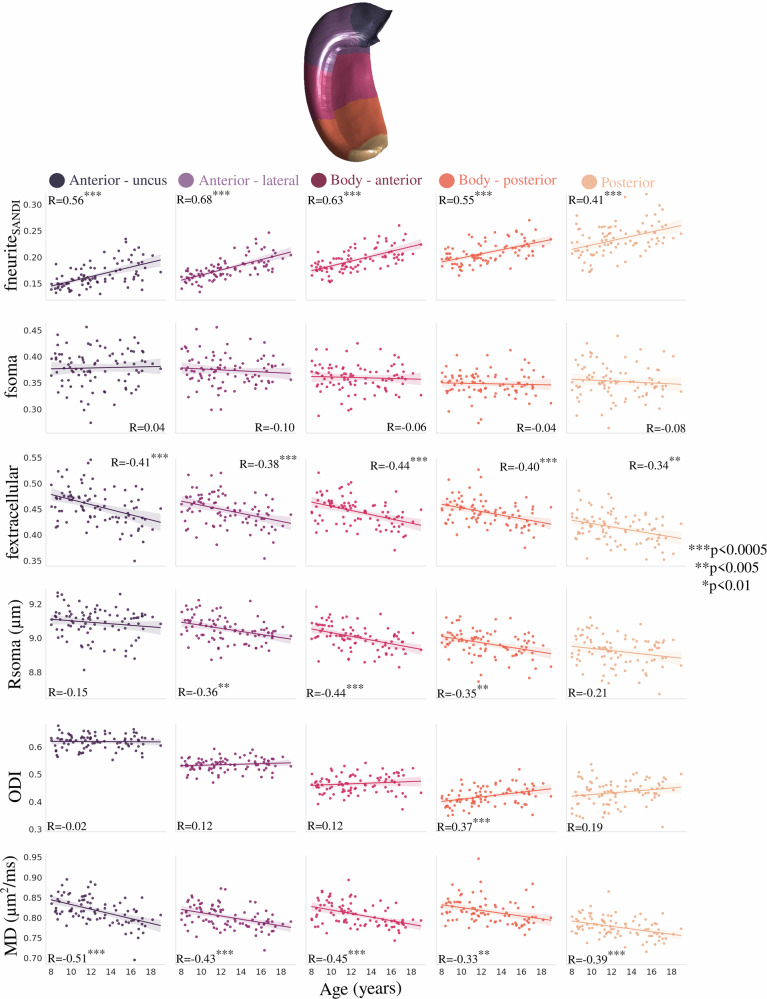
Correlation between age and anterior-posterior averaged microstructural measures. Colors represent hippocampal long-axis parcellations (shown on a midthickness surface at the top), and relationships are quantified using Pearson’s correlation coefficient (*R*) with the significance represented by asterisk(s), which is the probability that two uncorrelated variables could produce a correlation similar to the observed correlation value. Translucent bands around the regression line show the 95% confidence interval based on 1000 bootstrap resamples of the data.

Figure 5 depicts the correlation between long-axis averaged microstructural measures and age. fneurite_SANDI_ was significantly positively correlated with age within each long-axis parcellation, while fextracellular and MD were significantly negatively correlated with age (Fig. 5). Rsoma was significantly negatively correlated with age in the lateral anterior and body portions of the hippocampus, while ODI was significantly positively correlated with age only in the posterior portion of the hippocampal body. Similar to fneurite_SANDI,_ fneurite_NODDI_ was significantly positively correlated with age across the long-axis, while FA was not significantly correlated with age in any long-axis parcel (Supplementary Fig. S6). Unlike within the subfields, the age-related long-axis slopes were not significantly different across the parcellations for fneurite_SANDI_ (*F*(4424) = 0.323, *p* = 0.862), fsoma (*F*(4424) = 0.292, *p* = 0.883), fextracellular (*F*(4424) = 0.373, *p* = 0.828), Rsoma (*F*(4424) = 0.804, *p* = 0.523), ODI (*F*(4424) = 2.054, *p* = 0.086), and MD (*F*(4424) = 1.126, *p* = 0.344) (Supplementary Table S4).

### Diffusion orientation differences across age

**Fig. 6:**
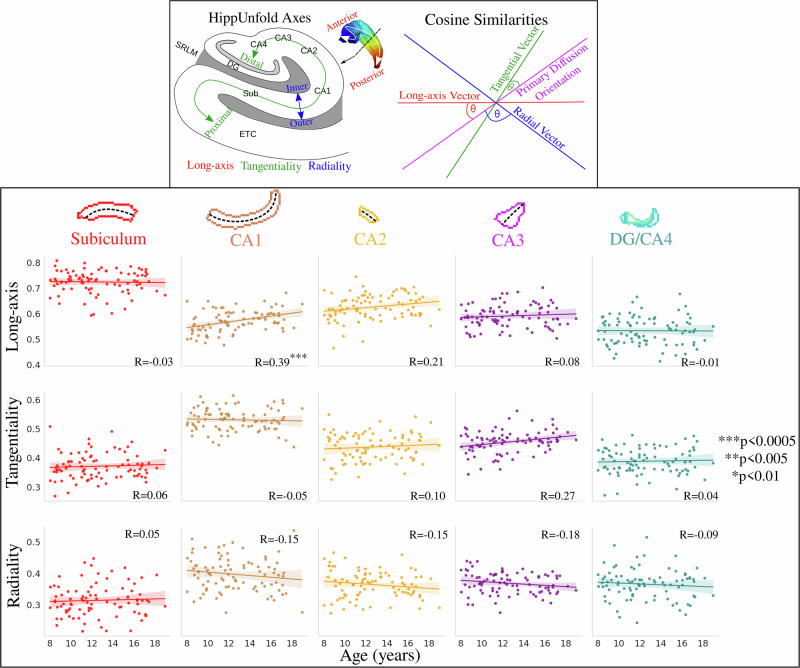
Relationship between age and subfield-averaged diffusion orientations. Top box (adapted from Karat et al.^89^) depicts the calculation of the cosine similarity, where the HippUnfold axes are used to generate long-axis, tangential, and radial vectors which are then compared with the primary diffusion orientation (peak 1 from the fiber orientation distribution function). Translucent bands around the regression line show the 95% confidence interval based on 1000 bootstrap resamples of the data.

Using the first peak of the fiber orientation distribution function (fODF) we quantified how diffusion orientations vary across age. Figure 6 depicts the correlation between age and subfield-averaged measures of long-axis, tangential, and radial oriented diffusion. Long-axis oriented diffusion was significantly positively correlated with age in CA1. The age-related slopes were not significantly different across the subfields for the long-axis orientations (*F*(4424) = 1.6, *p* = 0.173), tangential orientations (*F*(4424) = 1.125, *p* = 0.344), and radial orientations (*F*(4424) = 0.767, *p* = 0.547).

**Fig. 7:**
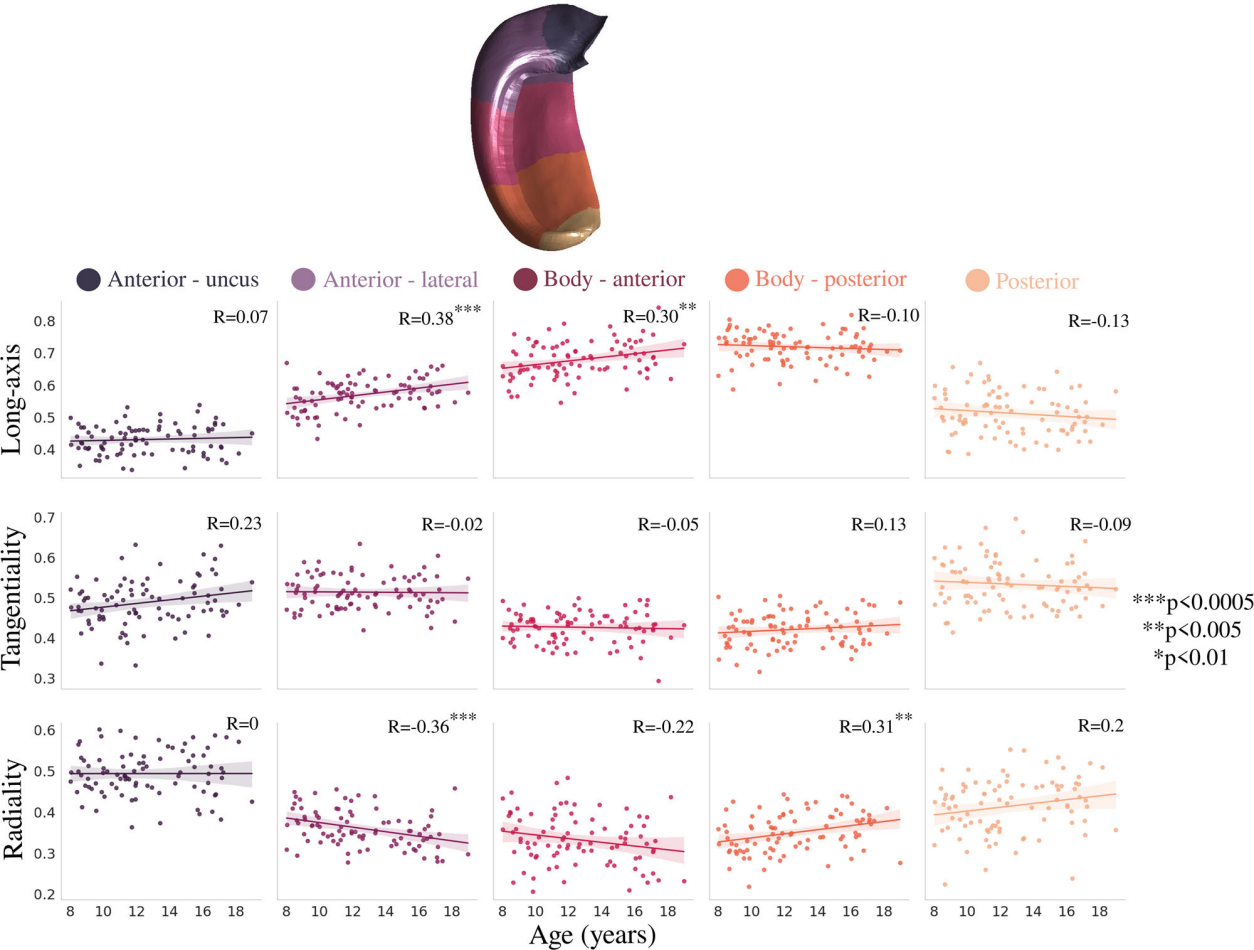
Relationship between age and anterior-posterior averaged diffusion orientations. Colors represent hippocampal long-axis parcellations (shown on a midthickness surface at the top). Translucent bands around the regression line show the 95% confidence interval based on 1000 bootstrap resamples of the data.

Figure 7 depicts the same diffusion orientation metrics averaged across the long-axis parcellation. Long-axis oriented diffusion was significantly positively correlated with age in the lateral anterior region and the anterior hippocampal body. Radial-oriented diffusion displayed a significant negative correlation with age in the lateral anterior region and a significant positive correlation with age in the posterior hippocampal body. This suggests that the diffusion orientation correlations with age are occurring more in the anterior and body of the hippocampus. Interestingly, unlike across the subfields, the age-related slopes in Fig. 7 were significantly different across the long-axis parcellations for both long-axis (*F*(4424) = 3.190, *p*-adjusted = 0.020) and radial (*F*(4424) = 5.341, *p*-adjusted = 0.001) oriented diffusion.

### Vertex-wise relation between macro- and microstructure across age and the hippocampal proximal-distal and long-axis

**Fig. 8:**
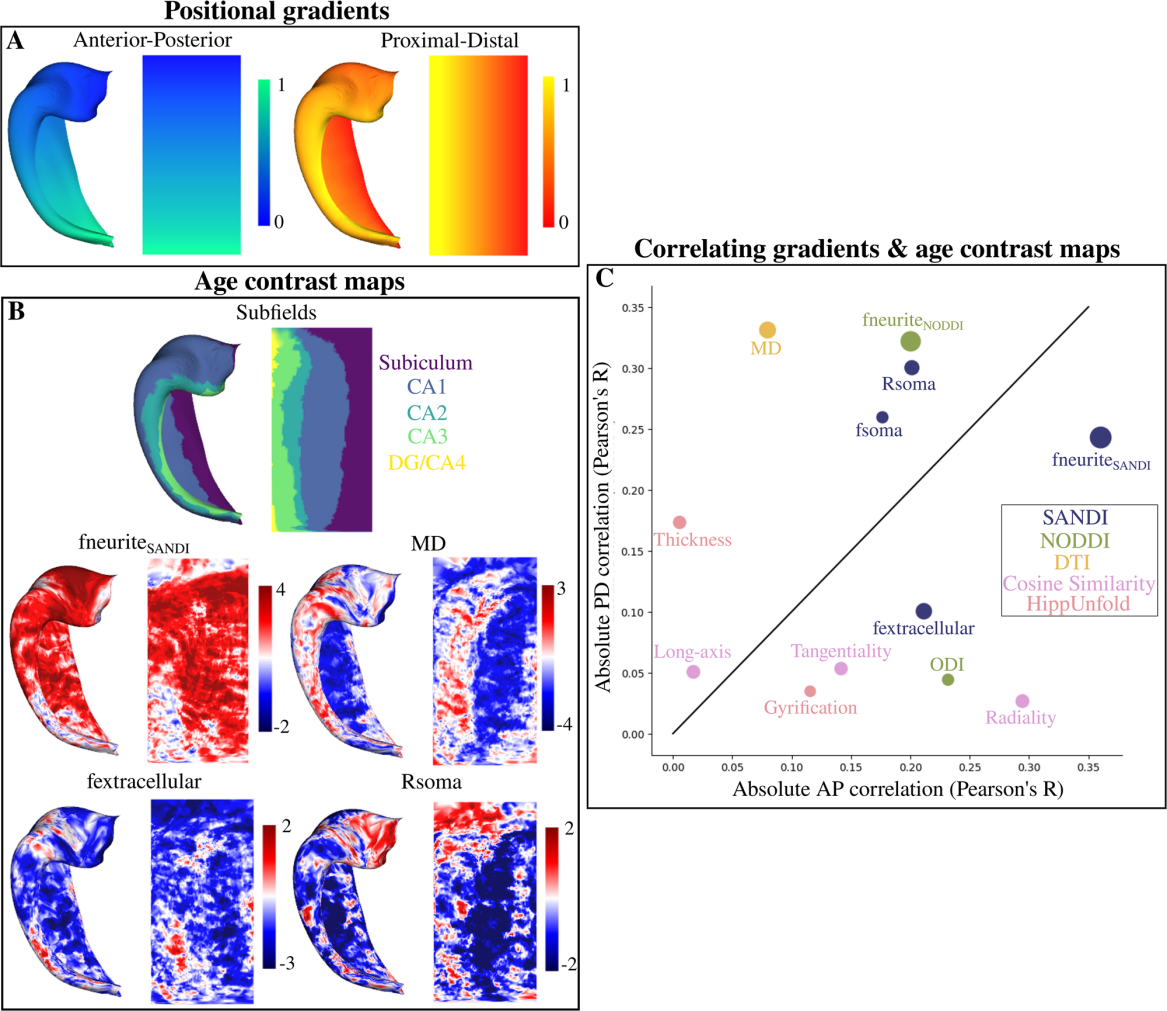
Correlating age-contrasted t-statistic maps with anterior-posterior (AP-long-axis) and proximal-distal (PD-subfields) gradients. **A** Gradients generated on a canonical surface across the AP and PD axis. **B** Subfield parcellation and four examples of a vertex-wise map with a contrast of age. The age-contrast maps capture the age-related trends of each metric at each vertex. Maps were calculated with hemisphere-averaged data and plotted on a left hippocampal surface. **C** Absolute correlation (Pearson’s *R*) of all the age-contrast maps (**B**) with the gradients (**A**). The size of the points represents the mean of the absolute values of the t-statistic across all vertices—a coarse measure for the total relationship between a metric and age. Color-coding refers to where each metric is derived from. Identity line is shown as a solid black line.

Surface t-statistic maps, which capture the spatial differences in macro- and microstructural across age were generated (Fig. 8B). These age-contrast maps were then correlated with contrived positional AP and PD gradients^39^ (Fig. 8A). The correlation then describes along which hippocampal axis are any age-related macro- or microstructural changes occurring (Fig. 8C). For example, the age-contrast map of MD (Fig. 8B) can be seen to vary largely along the PD axis. This then presents as a large PD correlation with a small AP correlation (Fig. 8C—greatly above the unit line). The size of the points in Fig. 8C represent the mean of the absolute values of the t-statistic across all vertices, which is a coarse measure for the total relationship between a metric and age. In line with Fig. 3, the absolute value of the t-statistic is largest for fneurite_SANDI_ (|*t*| = 2.58), fneurite_NODDI_ (|*t*| = 2.24), fextracellular (|*t*| = 1.41), Rsoma (|*t*| = 1.20), and MD (|*t*| = 1.52). The absolute value of the t-statistic is lower for thickness (|*t*| = 0.91), gyrification (|t| = 0.67), fsoma (|t| = 0.76), ODI (|*t*| = 0.76), and the long-axis (|*t*| = 0.97), tangential (|*t*| = 0.93), and radial (|*t*| = 0.99) oriented diffusion. Interestingly, the age-related increases of fneurite_NODDI_ and fneurite_SANDI_ (both approximately representing stick signal fractions) appear to be correlated relatively differently to the positional gradients. fneurite_NODDI_ is more correlated to the PD positional gradient (above the unit line) while fneurite_SANDI_ is more correlated to the AP positional gradient (below the unit line). Analyzing the cosine similarities, it can be seen that the diffusion orientations tend to vary more along AP across age.

The correlations between all the age-contrast maps (i.e., looking at metric-age covariance) is shown in Supplementary Fig. S8. Overall, there appears to be substantial correlation between many of the age-contrasted microstructural maps, suggesting that disparate metrics are capturing similar age-related microstructural differences.

### Correlations of vertex-wise microstructural differences across age with MRI, PET, and histology maps

**Fig. 9:**
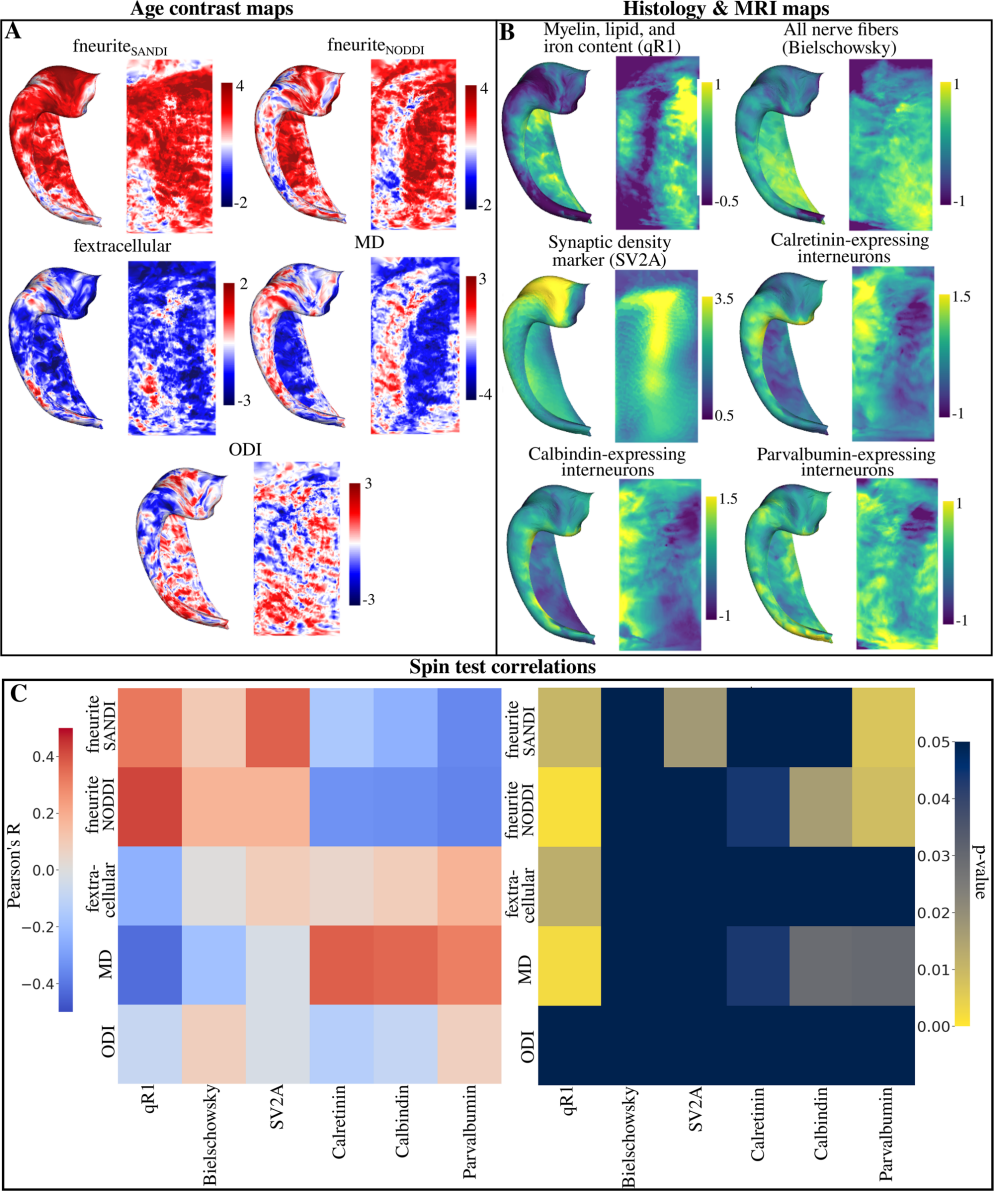
Correlating age-contrasted t-statistic maps with maps derived from histology, PET, and MRI. **A** Vertex-wise t-statistic maps with a contrast of age. The age-contrast maps capture the age-related trends of each metric at each vertex. Maps were calculated with hemisphere-averaged data and plotted on a left hippocampal surface. **B** Surface maps derived from histological staining, PET and MRI^39–46^. qR1, bielschowsky, calretinin, calbindin, and parvalbumin immunoreactivity maps were derived from^39,41^. The synaptic vesicle glycoprotein 2A (SV2A) marker was derived from^40,42,44,45^. **C** Left heatmap displays the Pearson’s *R* correlation between all age-contrast maps in (**A**) and all histology and MRI maps in (**B**). Right heatmap displays the uncorrected *p*-values derived from a hippocampus spin test using 2500 permutations^89^. Note that the color bar is inverted such that any brighter component of the heatmap corresponds to a significant *p*-value.

The same age-contrast maps in the above section (Fig. 9A) were used to correlate with maps from histology, PET, and MRI at high resolutions (Fig. 9B) derived from previous research^39–46^. The goal of this analysis was to contextualize the age-related differences in dMRI-derived microstructural measures. For example, it may be predicted that the increased fneurite_SANDI_ across age will be greatest in regions of higher adult myelin content or synaptic density as this reflects the spatial location of potential restrictions to diffusion. Briefly, we correlated the age-contrast maps with maps of quantitative R1 (qR1)-(myelin, lipid, and iron content)^39,41^, Bielschowsky staining (all nerve fibers)^39,41^, synaptic vesicle glycoprotein 2A (SV2A-synaptic density)^40,42,44,45^, and immunolabelling of calretinin, calbindin, and parvalbumin (expressing interneurons)^39,41^. Figure 9C and the below paragraph presents the correlation and uncorrected p-values between age-contrast maps (Fig. 9A) and all histology, PET, and MRI maps (Fig. 9B).

The age-related differences in fneurite_SANDI_ were positively correlated with qR1 (*R* = 0.33, *p* = 0.01) and SV2A (*R* = 0.37, *p* = 0.017) and negatively correlated with parvalbumin immunoreactivity (*R* = −0.36, *p* = 0.007). The age-related differences in fneurite_NODDI_ were positively correlated with qR1 (*R* = 0.42, *p* = 0.002) and negatively correlated with calretinin (*R* = −0.34, *p* = 0.04), calbindin (*R* = −0.34, *p* = 0.02), and parvalbumin immunoreactivity (*R* = −0.36, *p* = 0.007). The fextracellular age-contrast map was negatively correlated with qR1 (*R* = −0.24, *p* = 0.012). Age-related differences in MD were positively correlated with calretinin (*R* = 0.38, *p* = 0.044), calbindin (*R* = 0.36, *p* = 0.029) and parvalbumin immunoreactivity (*R* = 0.31, *p* = 0.030) and negatively correlated with qR1 (*R* = −0.42, *p* = 0.002). The correlation of ODI and age was found not to correlate strongly with any histology or MRI map. With FDR correction, only the correlations between fneurite_SANDI_ and qR1, SV2A, and parvalbumin immunoreactivity, fneurite_NODDI_ and qR1, calbindin, and parvalbumin immunoreactivity, and MD and qR1 remained significant. Finally, we found no correlation between Merker cell body staining^43^ and the fsoma age-contrast map (*R* = 0.04, *p* = 0.55) and the fsoma map averaged across age (*R* = 0.14, *p* = 0.30) seen in Supplementary Fig. S9.

## Discussion

We probed age-related alterations of hippocampal soma and neurite microstructure using a recent approach for surface-based hippocampal modeling. We found no significant age-related differences in volume, gyrification, and thickness, while significant age-related differences in microstructure measures related to neurites, soma, and MD were found. Sex-specific differences in age-related differences to macro- and microstructure were also found. Leveraging the salient orientation information derived from dMRI, localized diffusion orientation shifts across the hippocampal subfields and long-axis were uncovered, which may relate to the specific development of the intrahippocampal circuitry. Surface-based analyses suggested large variation in age-related microstructural differences across the proximal-distal (PD) (subfield) and AP (long-axis) axes, which may reflect ostensible developmental differences along these two axes. Finally, correlation of the age-contrasted microstructure surface maps with MRI, PET, and histology allowed for postulation of the potential underlying microstructure that age-related differences in diffusion may be sensitive to.

### Microstructure is strongly correlated with age across the subfields and long-axis

Recent evidence has suggested that the hippocampus undergoes a prolonged period of both structural and functional development after birth^47^. To investigate hippocampal microstructural changes across development, most previous research has used DTI^27^. In the current study, we found MD significantly decreased with age across the subfields and long axis. At the whole hippocampus level, Callow et al.^10^ found that MD decreased between 4 and 8 years of age. Langnes et al.^14^ found a protracted period of decreased MD in the anterior hippocampus up until 40 years of age, while posterior MD did not change much in the same range. Within a similar age range, Solar et al.^17^ found decreased MD in the anterior and posterior hippocampus from 5 years until around 40 years of age. In the current study, the decrease in MD across age was significant in both the anterior and posterior hippocampus. The decrease in MD is likely attributable to an increase in stick-like restrictions, as suggested by the significant increase across age in fneurite_SANDI_ and a corresponding decrease in fextracellular. This could correspond to increased myelination, proliferation of glia, increased dendrite branching and synaptic density, or other potential changes in restricted diffusion.

We found no significant difference in FA from 8 to 19 years (Supplementary Fig. S6), which conflicts with recent work. Vinci-Booher et al.^20^ found subfield-specific changes in FA across the age range of 5–30 years. In CA1, FA had an apparent parabolic trend across the whole age range. However, the trend of FA in CA1 appeared to decrease linearly when only considering ages 8–19. The DG and subiculum had a reduction in FA across the whole age range, while CA2/3 had an increase in FA^20^. Given that we found no significant difference in ODI and a significant increase in fneurite_SANDI_ across age, it may be hypothesized that FA should increase. However, it appears that FA is much more sensitive to changes in ODI than intraneurite signal fractions (Zhang et al.^38^). As well, the range of ODI (0.4–0.6) and fneurite_SANDI_ (0.15–0.30) values found in the current study can be seen to be in a regime of “low” FA contrast (Zhang et al.^38^). Thus, it appears that the hippocampal microstructural environment is generally isotropic at 8 years, and the increase in fneurite_SANDI_ is likely coming from an increase in spatially isotropic stick-like restrictions (which ostensibly would not change the already high ODI and thus result in no change in FA). Future investigation of age-related changes in FA is warranted.

Analyzing the primary orientation of diffusion across age, we found an increase in the long-axis oriented diffusion in the anterior lateral and body of the hippocampus, with a general corresponding decrease in the radial oriented diffusion. These differences may correspond to the development of intra-hippocampal pathways with known orientation. The fimbria is a white matter (WM) pathway, which sits atop CA3/CA4 and traverses the hippocampal long-axis, where, at its most posterior becomes the fornix^48^. The increase in long-axis oriented diffusion, which appears to largely occur in the anterior and body of the hippocampus, may be capturing changes to the coherent fimbria pathway. However, if this was the case, we may also expect to see a decrease in ODI and a corresponding increase in FA in the same region. The increase in long-axis oriented diffusion in these regions could also correspond to a change in the perforant path, which at some levels is oriented across the long-axis^48^

### Microstructure appears much more sensitive to age-related differences then macrostructure

One of the most common metrics used to investigate in vivo hippocampal development is volume (in mm^3^) derived with structural MRI. While common, there are conflicting results related to hippocampal volume changes in childhood and adolescence. We found no significant difference in volume for any long-axis parcel or subfield (measured using HippUnfold and FreeSurfer) between 8 and 19 years. At the level of the whole hippocampus, some studies have reported an increase in volume in late childhood and adolescence^13,18,21^ while others have found a very slight increase or no volume change in the same age range^11,19,49–51^. Coupé et al.^49^ analyzed 2994 subjects across the whole lifespan and found a very fast whole hippocampal volume increase until 8–10 years, followed by a very slow volume increase until 40–50 years. However, the hippocampus is not a monolithic structure, rather, its sub-regions ostensibly have different developmental trajectories. Langnes et al.^14^ found a relative increase in both anterior and posterior hippocampal volumes from 4 to 20 years, while Gogtay et al.^12^ found posterior volumes increased while anterior volumes decreased between 4 and 25 years. Across all the subfields, Krogsrud et al.^13^ found an increase in volume from ages 4 to 22 years, with an asymptote occurring around age 16 for all subfields. Contrastingly, Tamnes et al.^18^ found an initial slight increase and then slight decrease in volume in the subiculum and CA1 between ages 8 and 30. While not significant, we did see a slight decrease in the volume of the subiculum between 8 and 18 years of age (*R* = −0.16). Linear volume decreases were found in CA2/3, CA4, and the DG granular cell layer across the same age range.

Beyond volume, we also analyzed macrostructural measures of both thickness (in mm) and gyrification. Similar to the result for volume, we found no significant age-related differences in subfield and long-axis thickness and gyrification. This suggests that the general macrostructural form of the hippocampus may stabilize in early childhood. Although, notable differences exist between the current study and previous research. While macrostructure does not appear to change much after 8 years, we showed distinct microstructural differences between 8 and 19 years, suggesting that much of the age-related differences are internal to the hippocampal GM (and thus may be invisible to volume measurements). Diffusion in the hippocampus appears to provide improved sensitivity and specificity to hippocampal development then macrostructure^10^.

### Males and females display variable macro- and microstructural trends across age

Previous research has found varying trends in both volume and microstructure between males and females across age. We found that the age-related trends of volume and thickness were significantly different between males and females across the hippocampal subfields. In general, it appeared that females had no significant difference in volume and thickness from 8 to 19 years, while males had an increase in volume and thickness, particularly in CA1, CA2, and CA3. This may be explained by previous research, which suggests that female hippocampal volumes reach a plateau sooner than male volumes^16,19,51^. However, the evidence for the age at which this plateau occurs (and if it occurs at all) is conflicting. Giedd et al.^11^ found that hippocampal volume increased between ages 4 and 18 years only in females. Pfluger et al.^16^ found that hippocampal volumes increased much faster in females than in males between 1 month to 15 years of age. That is, at around 2 years of age, female hippocampal volumes plateaued, while male volumes kept increasing. This was corroborated by Uematsu et al.^19^ and Hu et al.^51^, where it was shown that females reach their peak hippocampal volume sooner than males. Contrastingly, Tamnes et al.^18^ found no sex differences in hippocampal subregion development between 8 and 30 years of age, and they found general volume changes in females across the whole age range (i.e., female volumes did not plateau in some subfields).

Few studies have examined sex differences in microstructural development of the hippocampus in late childhood and adolescence. In the current study, we found significant sex by age interactions in fneurite_SANDI_ and fextracellular, Rsoma, ODI, and MD, suggesting that the differences in microstructure across late childhood and adolescence are different between males and females. Vinci-Booher et al.^20^ found a nonlinear interaction of FA between sex and age in CA2/3. Callow et al.^10^ found sex was not significantly related to hippocampal MD. Interestingly, studies probing glia and neuron density and morphology in the Macaque hippocampus found no significant differences between males and females from juvenile to geriatric age, or from birth up to 1-year^52,53^. However, these studies were limited to small sample sizes, which makes any strong conclusion difficult.

It is highly likely that sex differences in hippocampal microstructure across age found here are driven by puberty, which is especially relevant given the earlier onset of puberty in females^54,55^. It is known that sex steroids (such as testosterone and estradiol) are capable of influencing brain organization, and that sex steroid availability during puberty could trigger structural reorganization of GM^56^. Corroborating this idea, in males testosterone was positively correlated with FA, while in females FA was negatively correlated with estradiol, suggesting that puberty and sex steroid availability can impact WM microstructure^57^. Ostensibly, sex differences in the current study could be related to dynamic changes in brain organization, given by puberty hormone changes. Furthermore, variability of the microstructure measures at a particular age within each sex could be related to differences in pubertal development. Though it was found that age correlated strongly with a self-reported measure of physical pubertal development (*R*^2^ = 0.72; Genc et al.^36^). While age is likely a good approximator of underlying physical development, there is likely large variability in sex steroid availability, which is not captured by age, which could be an additional factor affecting microstructure within each sex and age group. Future research is needed to further understand the developmental differences of hippocampal microstructure between males and females in the formative period of late childhood and adolescence, particular in relation to sex hormones and the onset of puberty.

### Age-related microstructural differences correlate to hippocampal axes and specific histological metrics

The hippocampus is generally studied in the context of subfields, which are the structurally distinct subunits of the hippocampus^1,30^. Recently, the hippocampal long-axis (anterior to posterior) has garnered substantial interest, given evidence that it is also structurally and functionally distinct^8,58–60^. Here, we investigated the age-related differences in microstructure within the context of the subfields and long-axis at the vertex level. We showed that age-related decreases in MD were much more correlated with the PD (i.e., subfields) than the long-axis, suggesting that the subfields have greater age-related variability from the perspective of diffusivity (Fig. 8). Previous research has found strong associations of age with MD particularly in the subiculum and CA1, where it does appear that age-related decreases in MD vary more across the subfields than the long-axis^61^. However, it has also been shown that the anterior hippocampus has an extended period of decreasing MD across late childhood and adolescence, while the posterior hippocampus remained relatively unchanged^14^. Interestingly, age-related increases in fneurite_SANDI_ varied more across the long-axis than the subfields, while Rsoma and fsoma showed trends similar to MD.

To further contextualize the results, we correlated adult MRI, PET, and histology maps of specific microstructural features with the age-related diffusion microstructure maps^39–46^. Interestingly, we found that age-related increases in fneurite_SANDI_ correlated with qR1 (myelin, lipids, and iron content) and a marker of synaptic density, SV2A. Put differently, in regions of high adult qR1/synaptic density, fneurite_SANDI_ increased across age. Similarly, we found that the age-related decreases in MD correlated with qR1, such that in regions of high adult qR1, MD decreased with age, and vice versa. The age-related differences in fneurite_SANDI_, fextracellular and MD thus may be related to myelin alterations in the hippocampus. Previous research has shown that even at 11 years of age, the density of myelinated fibers in humans did not reach adult levels, suggesting myelination continues through and beyond late childhood/adolescence^62^. Similarly, recent work has shown that oligodendrocyte-specific gene expression increased with age, indicating subsequent myelination processes^36^. Occurring concurrently with myelination is apparent glia alterations. In macaques, it was found that astrocyte process length and complexity increased from juvenile to adulthood^52^. An increase in stick-like glia processes across age may also ostensibly change fneurite_SANDI_. As well, the correlation of SV2A with fneurite_SANDI_ may be a result of increased dendrite ramifications. Indeed, Mellström et al.^63^ found a quadratic relationship between spine density and branching order of the basal dendrites of CA1 pyramidal neurons, suggesting that as branching order increases (more “sticks” from the diffusion perspective), there is higher spine density, and thus a greater synaptic density. Finally, in the current study, we found minimal differences in fsoma across age, and that it was not correlated to a Merker stain for cell bodies. Jabès et al.^53^ found that the number of principal neurons in the Macaque hippocampus did not change significantly from birth to 5–9 years of age in all subfields except the granule cell layer of the DG, which we did not have the resolution to isolate exclusively in the current study. It should be noted that the hippocampus of nonhuman primates is more developed at birth then the human hippocampus, and that the macaque is considered to be in adulthood at ages of 5–9 years. The measure of fsoma may not be expected to correlate with a stain for cell bodies given it’s a signal fraction rather than a volume fraction, as it does not account for T2 differences between microstructural compartments. The apparent decrease in Rsoma across age could be potentially related to an increase in glia presence. Findings from Genc et al.^36^ revealed that genes expressed in excitatory neuronal populations and oligodendrocytes increased over childhood and adolescence, whereas genes expressed in endothelial cells, astrocytes, microglia, and oligodendrocyte progenitor cells decreased in expression over childhood and adolescence. Furthermore, the contemporaneous increase of oligodendrocyte cell-type expression with in vivo MRI findings of decreased soma radius and increased neurite fraction across development may suggest that a higher proportion of smaller-radii cells occupied the space of the voxels studied across age.

It is likely that the microstructural correlations with age observed in the current work are related to an increase in cognitive capacity. Childhood to adolescence is a formative developmental period with many cognitive changes. In nonhuman primates it has been shown that synaptic density shortly after birth is higher than adult levels^22–24^. As well, the ability to perform a delayed-response task (a measure of working memory) coincides with the period, which marks the end of the highest synaptic density (~4 months of age in the Rhesus Macaque), linking microstructure with cognitive development^24^. In humans aged 4–8 years the MD of the hippocampus was found to decrease with age, and improved performance on a source memory task was associated with lower MD^10^. As well, it was found that age was a significant predictor of source memory, as older children performed better on the task^10^. Similarly, across an age range of 4–93.4 years, it was found that both volume and MD of the hippocampus showed age-dependent relationships with episodic memory, suggesting that the function of memory improves or declines with macro- and microstructural changes in the same age range^14^. While we did not measure memory or other cognitive outcomes, we posit that the microstructural differences seen here support the known increase in cognitive capacity between childhood and adolescence. For example, the significant increase across age in fneurite_SANDI_ can be attributed to an increase in stick-like restrictions ostensibly related to changes in the intrahippocampal circuitry which supports cognitive functions such as episodic memory.

### Limitations

One limitation of the current work was the 2 mm isotropic diffusion image resolution. Given the thickness of the hippocampus, some partial voluming with surrounding CSF and extra-hippocampal WM was expected. However, we attempted to minimize partial voluming by sampling the diffusion measures along the middle of the hippocampal GM. To examine how effective this was, we used FSL’s FAST tool to generate tissue type probabilities at the higher T1w resolution. We found relatively low CSF and WM (apart from the subiculum) tissue probability, suggesting that the sampling across the midthickness surface was mostly within the GM. Typically, high-resolution T2-weighted images are used for the segmentation of the hippocampal subfields in vivo^64,65^. In the current study, we did not have T2w data and thus relied on the T1w images for segmenting the subfields. However, we note that the segmentation of the hippocampal GM and its topological boundaries using the U-net provided in HippUnfold has been shown to be comparable between T1w and T2w data^37^. It should be noted that the U-net used in the current study was one provided directly in the HippUnfold toolbox, which was trained on adult T1w data^37^, which may not generalize well to the children and adolescent data used here. To this end, each U-net segmentation of the current study was extensively quality controlled and confirmed to provide a good segmentation of the GM and topological boundaries (examples shown in Supplementary Figs. S10 and S11). Based on the GM segmentations, the subfields can be propagated from a histological atlas to each individual subject. However, there is wide disagreement on how the subfields should be segmented, even with the availability of histological data^66,67^. Overall, given that the subfields are defined via cellular composition, obtaining such segmentations from lower resolution in vivo MRI is a fundamental challenge and an active area of research^64–67^. Similarly, there is inherent variability in contemporary methods, which seek to provide measures of hippocampal subfield volume through segmentation (HippUnfold, FreeSurfer, ANTs, manual delineation, etc.). Given this variability, it can be difficult to compare results pertaining to subfield and long-axis volume across studies. However, in the current study, both HippUnfold and FreeSurfer provided converging evidence that hippocampal volume did not change.

Another limitation was the correlation of the age-related microstructure t-statistic maps with static maps derived from adult histology. First, this correlation is across demographic variables and include different subjects. As well, the histology, MRI and PET measures used in the current study for correlation are known to change across age. This is particularly relevant for the SV2A measure, as synapses are dynamic across age in the hippocampus. Similarly, the number of calbindin and parvalbumin immunoreactive neurons have been found to change greatly across age in the hippocampus, though the calbindin immunoreactivity was found to reach adult-like levels at eleven years of age^68,69^. As well, the calretinin immunostaining has been shown to be greatly affected by subject age, fixation procedure, and postmortem delay^70,71^. The postmortem delay of the data used in the current study was less than 24 h^41^, which could ostensibly affect the quality of the calretinin immunoreactivity. The analysis of the current study can provide some insight into the underlying adult microstructure that is present in regions where diffusion-derived changes are occurring. However, further analysis with histology-derived age-contrasted maps will be required to relate diffusion changes across age to the true underlying changes in the cellular architecture, which may not be feasible to collect with sufficient power.

The current study had a moderate sample size at 88 participants with a mean age of 12.6 years. That is, there were more samples in the younger 8–12 year age range then the older 14–19 year range, which could make the results more reflective of changes occurring mainly in the late childhood stage. Given some of the strong correlations found in the current study, it would be valuable to examine the same advanced diffusion metrics across the whole lifespan instead of the narrower slice of childhood and adolescence. Likewise, it would be useful to probe diffusion changes longitudinally between childhood and adolescence, rather than the cross-sectional design used here.

## Conclusion

The hippocampus serves multiple cognitive functions, yet little is known about its microstructural development. Here we report, for the first time, distinct neurite and soma developmental profiles in the hippocampus during late childhood and adolescence using advanced diffusion modeling. Specifically, we report an age-related increase in neurite fraction and concurrent decrease in extracellular fraction and soma radius, which appears to be subfield and long-axis specific. Future research should look to examine the same diffusion measures across the whole lifespan and correlate these with cognitive processes that rely on the hippocampus, such as memory and spatial navigation.

## Materials and methods

### Data acquisition and preprocessing

Eighty-eight participants aged 8–19 years (42 male, mean age = 12.6, SD = 2.9) were scanned on a 3T Siemens Connectom system with ultra-strong (300 mT/m) gradients^35,36^. Age was found to correlate strongly with a self-reported measure of physical development using the pubertal development scale^72^, with *R*^2^ = 0.72^36^. Thus, age is used here as a close approximator of underlying physical development. The children were recruited as part of the Cardiff University Brain Research Imaging Centre Kids study, approved by the School of Psychology ethics committee at Cardiff University. Written informed consent was obtained from the primary caregiver of each child participating in the study, and adolescents aged 16–19 years also provided written consent. All ethical regulations relevant to human research participants were followed. Children were excluded from the study if they had repeated history of major head injuries or epilepsy, or if they had any contraindication to MRI (i.e., metal implants). For further information on recruitment, the reader is referred to Genc et al.^36^. Structural T1-weighted (T1w) images were acquired at 1 mm isotropic resolution (TE = 2 ms, TR = 2300 ms). dMRI data were acquired at 2 mm isotropic resolution (TE = 59 ms, TR = 3000 ms) with *b*-values of 0 (14 volumes, interleaved), 0.5 (30 directions), 1.2 (30 directions), 2.4 (60 directions), 4.0 (60 directions), and 6.0 (60 directions) ms/µm^2^. Diffusion directions were determined using an electrostatic repulsion algorithm generalized across all shells^73^. Data were acquired using an AP phase-encoding direction, with one additional inverse phased encoding (posterior-anterior) volume. The total dMRI acquisition time was 16 min and 14 s.

Data pre-processing has been described previously in Genc et al.^35^. Briefly, preprocessing steps included: denoising^74^, slice-wise outlier detection^75^, drift correction^76^, motion, eddy, and susceptibility-induced distortion correction^77^, Gibbs ringing correction^78^, bias field correction^79^, and gradient nonuniformity correction^80^.

Code to reproduce the results is openly available^81^. Numerical source data underlying all figures can be found in the supplementary data file.

### Surface modeling with HippUnfold

An automated software (HippUnfold) for hippocampal subfield segmentation and surface-based mapping was used in this study^37^. HippUnfold uses a deep neural network (a “U-net”)^82^ to segment within each subject the hippocampal GM, dentate gyrus (DG), stratum radiatum lacunosum moleculare (SRLM), and the topological bounds of the hippocampus, including the hippocampal-amygdala transition area, medial temporal lobe cortex, pial surface, and the indusium griseum (Supplementary Fig. S10). Using the GM as the domain of interest and the previously defined hippocampal boundaries, Laplace coordinates are generated along the AP, PD, and inner-outer directions, which define a complete 3D coordinate system of the hippocampus, which respects subject morphology. Using these coordinates, it is then possible to project subfields defined from a histological atlas to each subject in their native space, as well as generate surfaces at varying inner-outer/laminar depths. The subfields include the subiculum, Cornu ammonis (CA) 1–4, the DG, and the SRLM. In the current work, the DG and CA4 were averaged together. As well, using the above coordinates and generated surfaces, we parcellated the hippocampal AP (also referred to as the long-axis) into 5 bins, including the anterior uncus, anterior lateral, body anterior, body posterior, and posterior/tail. For more detailed methods, see DeKraker et al.^37^.

All U-net segmentations were reviewed by author BGK for errors. The review process first involved ranking the U-net segmentations as a success or failure, with success being defined as a high-quality segmentation or a good segmentation that may benefit from manual correction. A failure was defined as a segmentation that could not be manually corrected or would need to be completely segmented manually. All segmentations reviewed were classified as a success. All segmentations were then ranked on a scale of 1–3, with 1 being a high-quality segmentation requiring no manual correction and 3 being a decent quality segmentation that could use some manual correction. Of the 176 hippocampi (88 participants with left and right hemisphere), 162 segmentations were rated a 1, 7 segmentations were rated a 2, and 7 segmentations were rated a 3. Thus, 14 U-net segmentations (those rated a 2 and 3) from 12 participants were manually corrected for small over- or underestimations of tissue (examples of some manual corrections are shown in Supplementary Fig. S11). Of the 14 U-net segmentations corrected, 8 were from the left hemisphere, and 6 were from the right hemisphere. The 12 participants with manually corrected segmentations had a mean (SD) age of 11.92 (2.87) years, with a range of 8.01–16.77 years, including 4 females and 8 males. The age distribution of those with manual correction was not significantly different than those without manual correction (*t*(86) = 0.81, *p* = 0.42). The proportion of males and females was also not significantly different between those with and without manual correction (*X*2(1, *N* = 88) = 2.0, *p* = 0.16). HippUnfold was rerun for these 12 participants using the manually corrected tissue segmentations.

The metrics from HippUnfold used in this study were gyrification and thickness, which are calculated on the generated surfaces, and the more traditional measure of subfield volume calculated in each subject’s native space. All other volumetric measures of interest (i.e., the microstructure maps) were sampled onto the midthickness (middle of the GM) surface for visualization and analysis. All midthickness surfaces used in this study were composed of 7262 vertices, roughly corresponding to a spacing of 0.5 mm. Connectome Workbench (https://github.com/Washington-University/workbench) was used to sample values at each surface vertex from volume data using the enclosing method. To investigate potential partial volume effects PVE, FSL’s FAST tool was used to generate partial volume tissue estimates of GM, WM, and cerebrospinal fluid (CSF) using the T1w images^83^. These partial volume maps were then linearly interpolated to the lower 2 mm isotropic diffusion resolution and sampled on the midthickness surface in the same way as the other metrics of interest. Analyses were then performed to examine if estimated tissue type probabilities of GM, WM, and CSF varied with age. Finally, to provide a complementary measure of subfield volume, FreeSurfer (version 7.2.0) was run across all subjects using the T1w images.

### Microstructural modeling

The dMRI data were analyzed using DTI^27^, NODDI^38^, and SANDI^34^. The FMRIB Software Library (FSL; version 6.0.5)^84^, was used to fit the diffusion tensor using the *b* = 0, 0.5, and 1.2 ms/µm^2^ data. DTI characterizes the diffusion process as a symmetric 3 × 3 tensor which describes diffusion in different directions, where the diffusion signal can be written as:

1 $${{\rm{S}}}={{{\rm{S}}}}_{0}{{{\rm{e}}}}^{ < -{{\rm{B}}},{{\rm{D}}}{ > }_{{{\rm{F}}}}}$$

where S is the diffusion signal along a particular direction, B is the b-matrix^85^, D is the diffusion tensor, and <*>_F_ is the Frobenius inner product. The eigendecomposition of D provides useful metrics which capture ensemble diffusion characteristics, including FA (the variance of the eigenvalues-preferential diffusion along a particular direction) and the MD (the mean of the eigenvalues-average diffusivity in physical units). While DTI has been extensively used to interrogate brain microstructure, the complex nature of water diffusion in neural tissue cannot usually be well described by a single tensor^86^.

The microstructure diffusion toolbox (MDT) (version 1.2.7)^87^ was used to fit the NODDI model using all *b*-values^38^. The NODDI model assumes that water diffusion is occurring in three non-exchanging microstructural environments consisting of intraneurite, extraneurite, and CSF compartments. The intraneurite compartment generally refers to the space enclosed by the membrane of neurites (assumed impermeable to water) and is modeled as a set of orientation dispersed sticks (i.e., cylinders with zero radius) according to the Watson distribution^38^. The extraneurite compartment comprises the space around neurites and is modeled as a cylindrically symmetric tensor. The parallel diffusivity of the intraneurite and extraneurite compartment was fixed to 1.7 µm^2^/ms. The CSF compartment is modeled as isotropic Gaussian diffusion described by a single diffusion coefficient, which was fixed to 3.0 µm^2^/ms. The diffusion signal according to the NODDI model can be written as^38^

2 $${{\rm{S}}}=\,({1-{{\rm{f}}}}_{{{\rm{iso}}}})({{{\rm{f}}}}_{{{\rm{in}}}}{{{\rm{S}}}}_{{{\rm{in}}}}+(1-{{{\rm{f}}}}_{{{\rm{in}}}}){{{\rm{S}}}}_{{{\rm{en}}}})\,+{{{\rm{f}}}}_{{{\rm{iso}}}}{{{\rm{S}}}}_{{{\rm{iso}}}}\,$$

where ${{{\rm{f}}}}_{{{\rm{iso}}}},{{{\rm{f}}}}_{{{\rm{in}}}},{{{\rm{f}}}}_{{{\rm{en}}}}$ is the signal fraction from the CSF, intraneurite, and extraneurite compartments, respectively, while ${{{\rm{S}}}}_{{{\rm{iso}}}},{{{\rm{S}}}}_{{{\rm{in}}}},{{{\rm{S}}}}_{{{\rm{en}}}}$ is the diffusion signal arising from the CSF, intraneurite, and extraneurite compartments, respectively. The two metrics from NODDI investigated in this study include the ${{{\rm{f}}}}_{{{\rm{in}}}}$, which will be referred to as fneurite_NODDI_, and the orientation dispersion index (ODI), which is derived from the Watson distribution.

To fit the SANDI model, the SANDI MATLAB toolbox (https://github.com/palombom/SANDI-Matlab-Toolbox-v1.0)^34,88^ was used. The SANDI model assumes that water diffusion occurs in the non-exchanging intraneurite, extracellular, and intrasoma compartments. Much like the NODDI model, the intraneurite compartment is represented as diffusion within sticks. The extracellular compartment is modeled as isotropic Gaussian diffusion characterized by a single diffusion coefficient. Finally, the intrasoma compartment is modeled as diffusion occurring in a restricting sphere with a radius ${{{\rm{r}}}}_{{{\rm{s}}}}$. The diffusion signal according to the SANDI model can be written as^34^

3 $${{\rm{S}}}=\,({1-{{\rm{f}}}}_{{{\rm{ec}}}})({{{\rm{f}}}}_{{{\rm{in}}}}{{{\rm{S}}}}_{{{\rm{in}}}}+(1-{{{\rm{f}}}}_{{{\rm{in}}}}){{{\rm{S}}}}_{{{\rm{is}}}})\,+{{{\rm{f}}}}_{{{\rm{ec}}}}{{{\rm{S}}}}_{{{\rm{ec}}}}\,$$

where ${{{\rm{f}}}}_{{{\rm{ec}}}}$ and ${{{\rm{f}}}}_{{{\rm{in}}}}$ are the signal fractions from the extracellular and intraneurite compartments, respectively, and ${{{\rm{f}}}}_{{{\rm{is}}}}=(1-{{{\rm{f}}}}_{{{\rm{in}}}})$ is the signal fraction of the soma compartment. ${{{\rm{S}}}}_{{{\rm{ec}}}},{{{\rm{S}}}}_{{{\rm{in}}}},{{{\rm{S}}}}_{{{\rm{is}}}}$ are the diffusion signals arising from the extracellular, intraneurite, and soma compartments, respectively. Beyond the addition of the soma compartment, the SANDI model focuses on estimating microstructural features that are orientation independent. That is, the diffusion signal is averaged across all gradient directions before model fitting. Thus, the direction-averaged formulations of ${{{\rm{S}}}}_{{{\rm{ec}}}}$ and ${{{\rm{S}}}}_{{{\rm{in}}}}$ depart from the functional forms of the signals in NODDI, which have an orientation dependence. Furthermore, unlike NODDI, SANDI fits the diffusivities of the intraneurite and extracellular compartments, while fixing the intrasoma diffusivity to 3.0 µm^2^/ms. The maps from SANDI analyzed in this study include fextracellular = ${{{\rm{f}}}}_{{{\rm{ec}}}}$, fneurite_SANDI_ = ${(1-{{{\rm{f}}}}_{{{\rm{ec}}}}){{\rm{f}}}}_{{{\rm{in}}}}$; fsoma =  ${(1-{{{\rm{f}}}}_{{{\rm{ec}}}}})(1-{{{\rm{f}}}}_{{{\rm{in}}}})$ and ${{{\rm{r}}}}_{{{\rm{s}}}}$ referred to as Rsoma.

Diffusion data and the corresponding metric maps were registered to each subject’s T1w space. Once aligned, all metrics of interest were sampled on the hippocampal midthickness surface. From the surface maps, metrics were then averaged within subfields or along the AP axis for further analysis.

### Orientation cosine similarities

Microstructure in the hippocampus tends to be aligned stereotypically along the AP (long-axis), PD (tangential), and/or inner-outer (IO; radial) axes^2,48,89,90^. Diffusion analysis can provide a fODF, where its peaks ostensibly correspond to the orientation of the underlying microstructure^91^. Similar to Karat et al.^89^, analyses were performed to examine how the first peak of the fODF was oriented relative to the three hippocampal axes, and how these orientations may shift across age. To calculate the fODF, we used the MRtrix3 toolbox (version 3.0.3)^92^ and multi-shell multi-tissue constrained spherical deconvolution (MSMT-CSD)^91^ using all *b*-values with a response function averaged across all subjects. We then extracted the first/largest peak from the spherical harmonic representation of the WM fODF (i.e., the orientation with the maximum signal change). To get an orientation measure along the three hippocampal axes, we obtained gradient vector fields along the AP, PD, and IO hippocampal axes by taking the first partial derivative of the respective Laplacian coordinates provided by HippUnfold along the x, y, and z spatial dimensions^89^

4 $$\begin{array}{c}\nabla {{{\rm{\psi }}}}_{{{\rm{AP}}}}({{\rm{x}}},{{\rm{y}}},{{\rm{z}}})=\left[\frac{\partial {{{\rm{\psi }}}}_{{{\rm{AP}}}}}{{\partial }_{{{\rm{x}}}}},\frac{\partial {{{\rm{\psi }}}}_{{{\rm{AP}}}}}{{\partial }_{{{\rm{y}}}}},\frac{\partial {{{\rm{\psi }}}}_{{{\rm{AP}}}}}{{\partial }_{{{\rm{z}}}}}\right]\\ \nabla {{{\rm{\psi }}}}_{{{\rm{PD}}}}\left({{\rm{x}}},{{\rm{y}}},{{\rm{z}}}\right)=\left[\frac{\partial {{{\rm{\psi }}}}_{{{\rm{PD}}}}}{{\partial }_{{{\rm{x}}}}},\frac{\partial {{{\rm{\psi }}}}_{{{\rm{PD}}}}}{{\partial }_{{{\rm{y}}}}},\frac{\partial {{{\rm{\psi }}}}_{{{\rm{PD}}}}}{{\partial }_{{{\rm{z}}}}}\right]\\ \nabla {{{\rm{\psi }}}}_{{{\rm{IO}}}}({{\rm{x}}},{{\rm{y}}},{{\rm{z}}})=\left[\frac{\partial {{{\rm{\psi }}}}_{{{\rm{IO}}}}}{{\partial }_{{{\rm{x}}}}},\frac{\partial {{{\rm{\psi }}}}_{{{\rm{IO}}}}}{{\partial }_{{{\rm{y}}}}},\frac{\partial {{{\rm{\psi }}}}_{{{\rm{IO}}}}}{{\partial }_{{{\rm{z}}}}}\right]\end{array}$$

where ${{{\rm{\psi }}}}_{* }$ is the Laplacian scalar field along one hippocampal axis which was obtained by solving Laplace’s equation: ${\nabla }^{2}({{{\rm{\psi }}}}_{* })=0$. The result of Eq. 4 is three vector images for each hemisphere and subject, which point along a single hippocampal axis (AP, PD, or IO). With the definition of the inner product between two vectors as ${{\rm{u}}}\cdot {{\rm{v}}}=\left|{{\rm{u}}}\right|\left|{{\rm{v}}}\right|\cos ({{\rm{\theta }}})$, we take these vector fields and the aligned first peak of the fODF and calculate voxel-wise cosine similarities as:

5 $$\begin{array}{c}{{\rm{Long}}}-{{\rm{axis}}}=\left|\left(\frac{{\overline{\nabla {{\rm{\psi }}}}}_{{{\rm{AP}}}}\cdot {\overline{{{\rm{fODF}}}}}_{{{\rm{peak}}}1}}{\left|{\overline{\nabla {{\rm{\psi }}}}}_{{{\rm{AP}}}}\right|\left|{\overline{{{\rm{fODF}}}}}_{{{\rm{peak}}}1}\right|}\right)\right|\\ {{\rm{Tangentiality}}}=\left|\left(\frac{{\overline{\nabla {{\rm{\psi }}}}}_{{{\rm{PD}}}}\cdot {\overline{{{\rm{fODF}}}}}_{{{\rm{peak}}}1}}{\left|{\overline{\nabla {{\rm{\psi }}}}}_{{{\rm{PD}}}}\right|\left|{\overline{{{\rm{fODF}}}}}_{{{\rm{peak}}}1}\right|}\right)\right|\\ {{\rm{Radiality}}}=\left|\left(\frac{{\overline{\nabla {{\rm{\psi }}}}}_{{{\rm{IO}}}}\cdot {\overline{{{\rm{fODF}}}}}_{{{\rm{peak}}}1}}{\left|{\overline{\nabla {{\rm{\psi }}}}}_{{{\rm{IO}}}}\right|\left|{\overline{{{\rm{fODF}}}}}_{{{\rm{peak}}}1}\right|}\right)\right|\end{array}$$

which is depicted in the top of Fig. 6. A cosine similarity of 0 corresponds to a case where the two vectors are orthogonal, while a cosine similarity of 1 corresponds to a case where the vectors are parallel. A higher cosine similarity thus relates to a case where diffusion is highly oriented along a particular hippocampal axis. A total of three scalar cosine similarity metrics were generated for each subject and hemisphere, and these metrics were sampled along the midthickness surface as described above.

### Statistics and data analysis

Multiple statistics were used to examine macro- and microstructure across age, sex, and hemisphere.

Pearson’s correlation coefficient was used to examine the relation between age and all macro- and microstructural measures at the subfield and AP averaged level. Using the pearsonr function in Scipy (version 1.11.3), hypothesis testing was performed to determine the probability that two uncorrelated variables could produce a correlation similar to the observed correlation value. Given the large number of exploratory correlations performed, we sought to set a minimum alpha for which the Pearson’s *R* would be considered significant. We set the minimum alpha to 0.01, which is analogous to a Bonferroni correction corresponding to 5 hypothesis tests (testing a metric across the 5 subfields). We also report thresholds of 0.005 and 0.0005 so that the correlations can be considered under a more conservative correction.

General linear modeling was used to examine the significance of the interaction between sex and age and hemisphere and age. That is, we sought to determine if the slopes of the macro- and microstructure metrics across age were significantly different between sexes and hemispheres. First, a full linear model was built containing all relevant terms, then a reduced model was built which contained all the same terms but removed a single variable of interest (sex and age or hemisphere and age interaction). An F-test was then performed between the full and reduced model to examine if the variable of interest resulted in a significantly better model (i.e. it captured a significant portion of variance in the dependent variable (DV)):

6 $${{\mathrm{LM}}}_{{\mathrm{reduced}}}= 	 \; {\mathrm{DV}} \sim {\,\beta }_{1}{\mathrm{age}}+{\beta }_{2}{\mathrm{subfield}}+{\beta }_{3}{\mathrm{sex}}+{\beta }_{4}{\mathrm{age}}:{\mathrm{subfield}}\\ 	 +{\beta }_{5}{\mathrm{sex}}:{\mathrm{subfield}}$$

$${{\mathrm{LM}}}_{{\mathrm{full}}}= 	 \; {\mathrm{DV}} \sim {\,\beta }_{1}{\mathrm{age}}+{\beta }_{2}{\mathrm{subfield}}+{\beta }_{3}{\mathrm{sex}}+{\beta }_{4}{\mathrm{age}}:{\mathrm{subfield}}\\ 	 +{\beta }_{5}{\mathrm{sex}}:{\mathrm{subfield}}+{\beta }_{6}{\mathrm{age}}:{\mathrm{sex}}$$

where: implies an interaction between two variables and the DV was a given macro- or microstructural metric. To examine AP differences, similar models were built by replacing the subfield term with an AP parcellation term instead. Note that for brevity, not all $\beta$f coefficients were shown here since each subfield and its interactions required its own coefficients. In this example, both models were identical except for an additional interaction term between age and sex. An F-test between the reduced and full model was then used to examine if the age by sex interaction term significantly improved the linear model. A significant F-test suggested that the change in the DV across age was significantly different between males and females. FDR correction was then applied to the p-values derived from the F-test across all metrics. For example, in Fig. 2 (corresponding to Eq. 6), we performed an FDR correction for 3 tests, corresponding to the same model being applied across 3 macrostructural metrics. In Fig. 4, we performed an FDR correction for 6 tests, corresponding to the same model being applied across 6 microstructural metrics. The same analysis was performed using an interaction of age and hemisphere, as well as age and subfield, with the same FDR correction as above.

The statistics mentioned thus far were applied only to the subfield and AP averaged values. To provide increased spatial fidelity of any age-related macro- or microstructural differences, statistics were also performed at the vertex level. At each vertex on the hippocampal surface a linear model was built of the form:

7 $${{\mathrm{LM}}}_{{\mathrm{vertex}}}={\mathrm{DV}} \sim {\,\beta }_{1}{\mathrm{age}}+{\beta }_{2}{\mathrm{sex}}+{\beta }_{3}{\mathrm{age}}:{\mathrm{sex}}$$

where the t-statistic for the contrast of age was extracted (where ${t}_{{\mathrm{age}}}={\,\beta }_{1}/{\mathrm{SD}}$ where ${\mathrm{SD}}$ is the standard deviation of${\beta }_{1}$). These age-contrasted t-statistic maps (referred to as age-contrast maps) capture the linear age-related differences of each metric at each vertex. The age-contrast maps were then correlated with contrived AP and proximal-distal positional gradient maps using Pearson’s *R* (Fig. 8A). The correlations with the gradients describe along which hippocampal axis any age-related microstructural differences align with.

### Correlations with MRI, PET, and histology

The age-contrast maps (described above) were correlated with high-resolution MRI, PET, and histology maps using a hippocampus spin test^89^. The hippocampal maps of qR1 derived from post-mortem MRI and histological measures derived from a bielschowsky (silver) stain, merker stain, and immunolabelling of calbindin, calretinin, and parvalbumin were obtained from previous research^41,93^ and incorporated in the open-source HippoMaps^39^ toolbox, which provides these measures already sampled along the midthickness surface of the hippocampus. The synaptic vesicle glycoprotein 2A (SV2A) PET marker was obtained from previous research^42,44,45,94^ and incorporated in the open-source NeuroMaps^40^ toolbox, where the same sampling as in the HippoMaps toolbox was performed on a hippocampal midthickness surface.

The maps of qR1, Bielschowsky staining, and immunolabelling of calbindin, calretinin, and parvalbumin were derived from previous work using a non-demented 75-year-old female and a 59-year-old female with both the left and right hemisphere included, thus totaling 4 hippocampi^41,93^. The presented maps were averaged across all 4 of the hippocampi. The post-mortem qR1 maps were obtained from previous work using a 7T MRI at 400 µm isotropic resolution and are thought to capture myelin, lipid, and iron content^41,93,95,96^. The Bielschowsky stain is representative of all types of nerve fibers^39,41,93,97^, while the calbindin, calretinin, and parvalbumin immunolabelling captures GABAergic interneurons (while calbindin is also present in excitatory DG granule cells), which express these proteins^39,41,93^. The maps of calbindin, calretinin, and parvalbumin depict the immunoreactivity of each of these proteins across the hippocampus. For more details about the histology and MRI processing we refer the reader to Alkemade et al.^41,93^.

The map of the merker histological stain was derived from the BigBrain^43,98^ dataset, which captures neuronal cell bodies. This includes the processing of the full brain of a 65-year-old male. For more information about the BigBrain datsaset we refer the reader to Amunts et al.^43,98^.

SV2A PET maps representative of synaptic density was derived from multiple in vivo datasets^42,44,45,94^, as incorporated in the NeuroMaps toolbox^40^. This data was averaged across 76 hippocampi (45 males) with a mean (SD) age of 48.9 (18.4) years. For more information, we refer the reader to the NeuroMaps toolbox^40^.

### Reporting summary

Further information on research design is available in the Nature Portfolio Reporting Summary linked to this article.

## Supplementary information

Supplementary Information

Description of Additional Supplementary Files

Supplementary Data 1

Reporting Summary

## Data Availability

The histology, ex vivo MRI, and PET maps can be found in both the HippoMaps (osf.io/92p34/) and NeuroMaps (https://github.com/netneurolab/neuromaps) toolbox. Due to the inclusion of minors (under 18 participants), the availability of derived or identifiable data from the participant cohort is restricted due to privacy concerns. Derived data supporting the findings of the imaging analyses are available by contacting the authors in writing via email, allowing 4 weeks for access requests to be granted. Numerical Source data underlying all graphs in this article can be found in the Supplementary data file.

## Code availability

The SANDI model was fit to the diffusion data using an open-source MATLAB toolbox (https://github.com/palombom/SANDI-Matlab-Toolbox-v1.0). Code to repeat the study analyses can be found at: 10.17605/OSF.IO/4AQXR.
