# Supplementary material for: Microstructural variation of hippocampal substructures across childhood and adolescence quantified with high-gradient diffusion MRI
Source: Commun Biol. 2026 Feb 12;9:416. doi: 10.1038/s42003-026-09622-x (PMC13009190; doi:10.1038/s42003-026-09622-x)
Supplement: Supplementary file 1 — Supplementary Information [file 42003_2026_9622_MOESM1_ESM.pdf]

Microstructural variation of hippocampal substructures across childhood and adolescence quantified with high-gradient diffusion MRI

Bradley G. Karat[1,2], Sila Genc[3,4], Erika P. Raven[4,5], Marco Palombo[4,6], Ali R. Khan[1,2,7], Derek K. Jones[4]

[1]Robarts Research Institute, Western University, London, ON, Canada; [2]Centre for Functional and Metabolic Mapping, Western University, London, ON, Canada; [3]Department of Neurosurgery, The Royal Children's Hospital, Melbourne, Australia; [4]Cardiff University Brain Research Imaging Centre (CUBRIC), Cardiff University, Cardiff, United Kingdom; [5]Center for Biomedical Imaging, Department of Radiology, New York University Grossman School of Medicine, New York, NY, United States; [6]School of Computer Science and Informatics, Cardiff University, Cardiff, United Kingdom; [7]Department of Medical Biophysics, Western University, London, ON, Canada

*Corresponding author: Bradley G. Karat, bradleykarat@gmail.com

This PDF file includes:

Figures S1 to S11
Tables S1 to S4

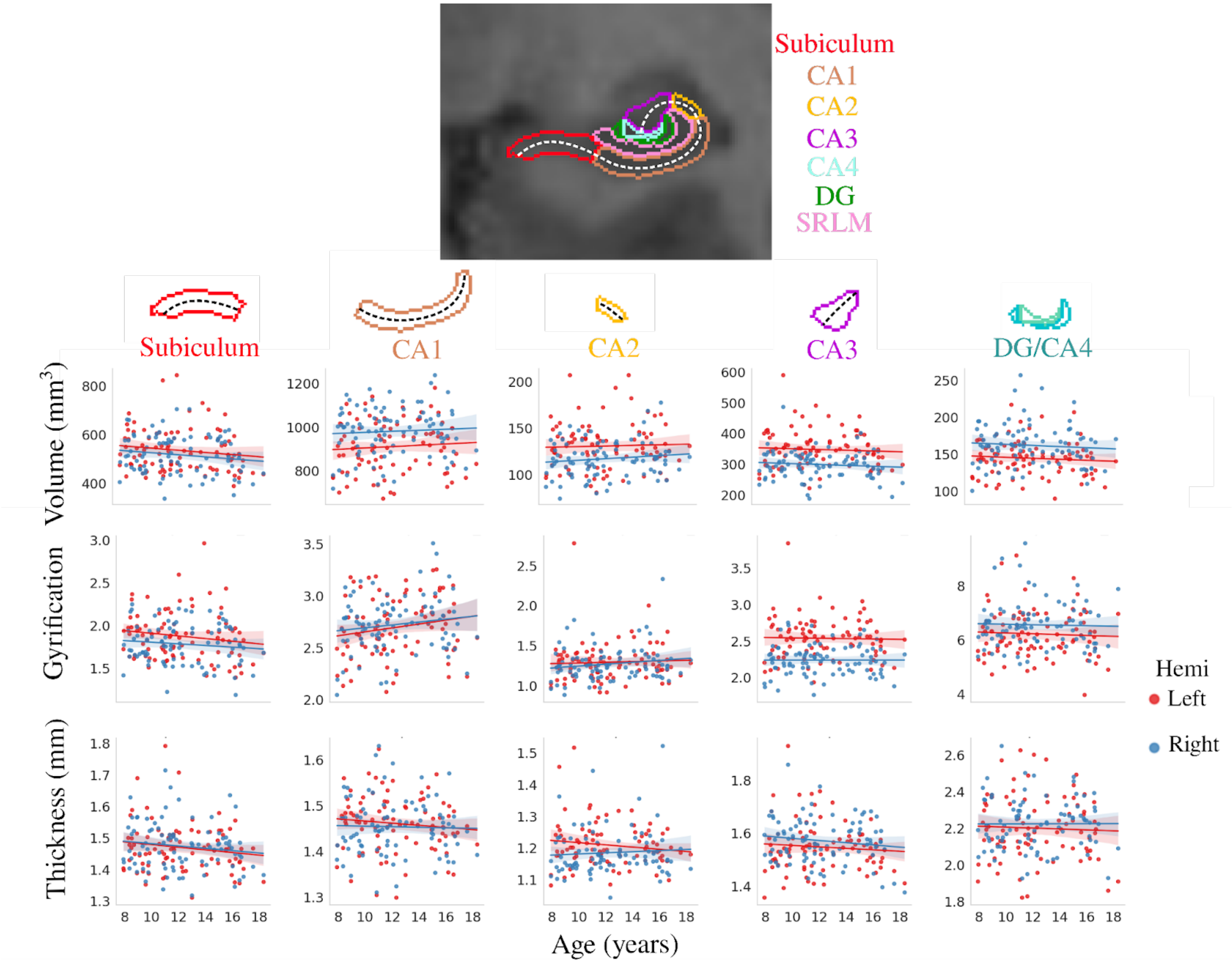

Supplementary Figure S1. Relationship between age and subfield averaged macrostructural measures of volume, gyrification, and thickness between the left and right hemisphere. The dashed lines approximately represent the midthickness surface which gyrification and thickness were calculated and then averaged on. CA - cornu ammonis; DG - dentate gyrus; SRLM - stratum radiatum lacunosum moleculare.

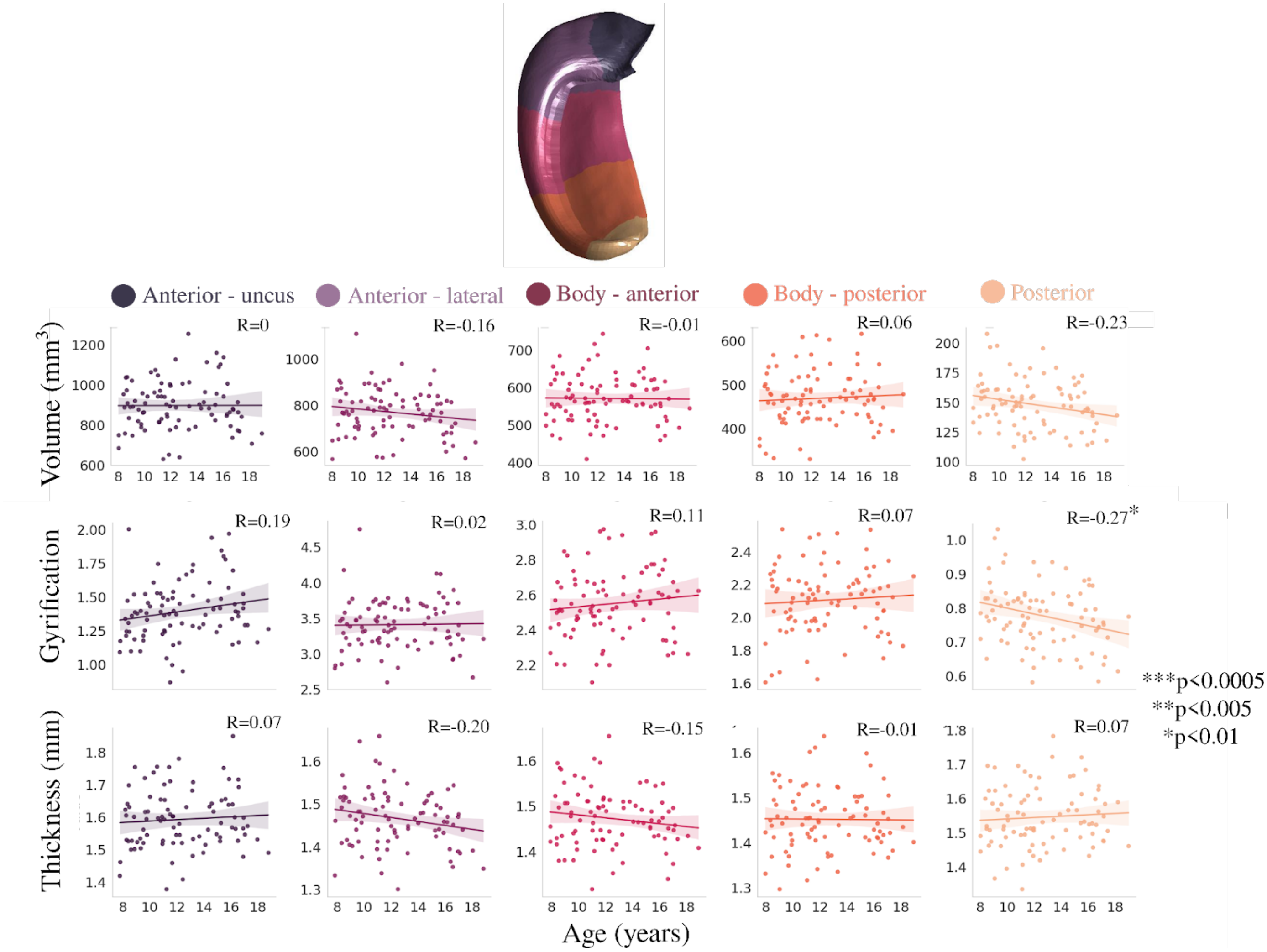

Supplementary Figure S2. Relationship between age and long-axis averaged macrostructural measures of volume, gyrification, and thickness.

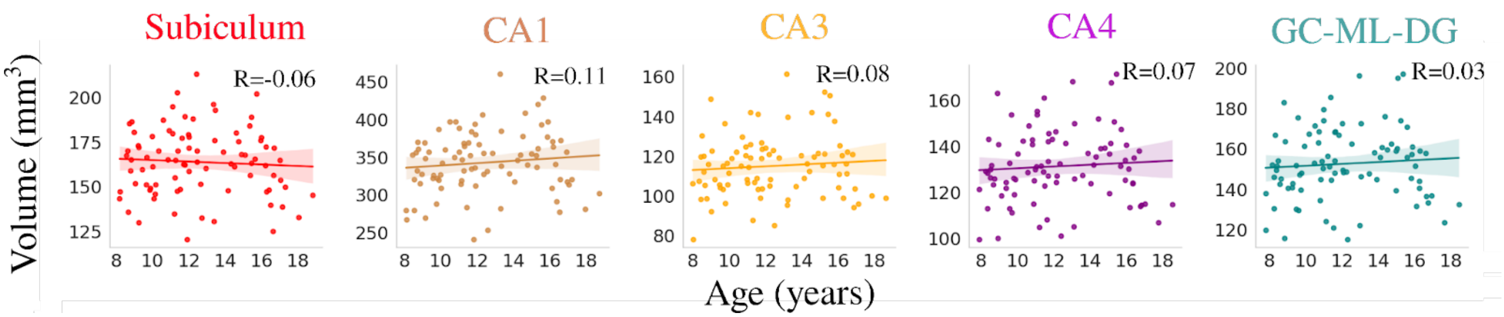

Supplementary Figure S3. Relationship between age and subfield volume derived from FreeSurfer.

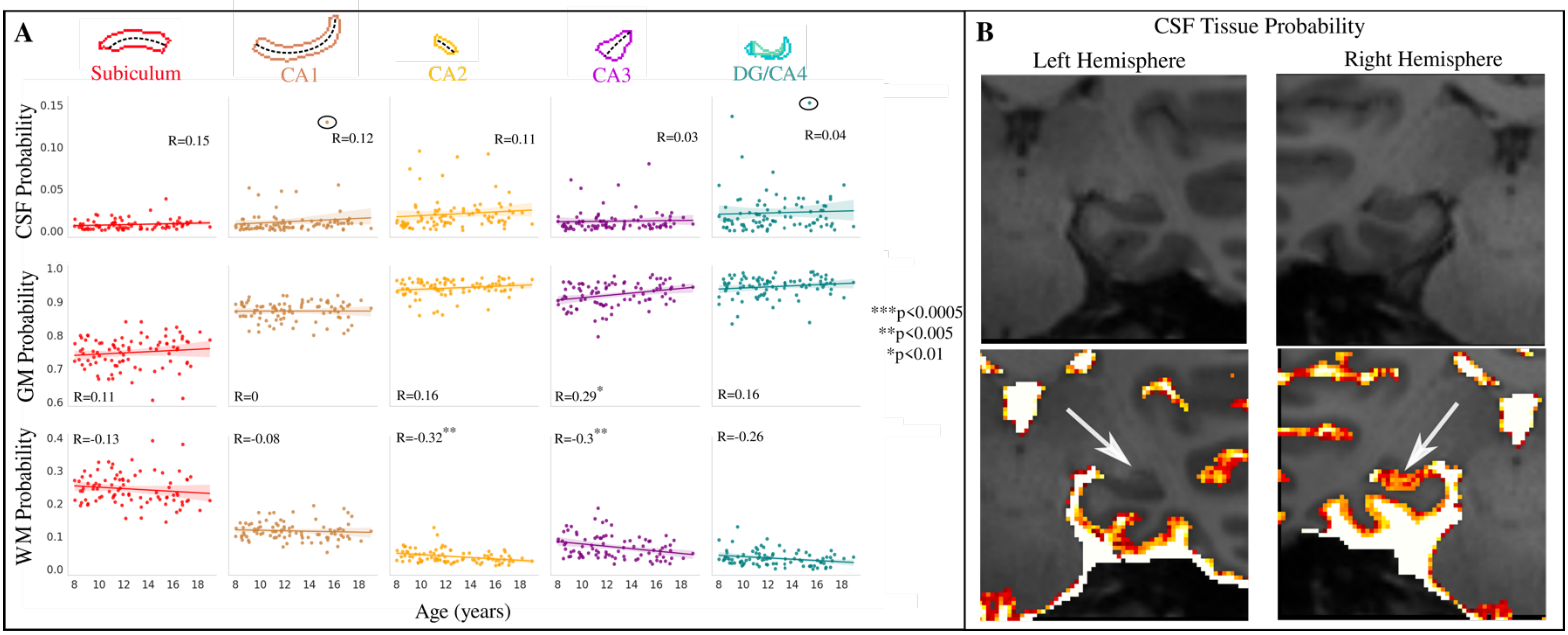

Supplementary Figure S4. (A) Relationship between age and subfield averaged partial volume measures of CSF, GM, and WM. The circled points represent a subject with a high probability of CSF. (B) T1w image and CSF probability output for the circled subject in (A). The right hemisphere has a misestimated high CSF probability. CA - cornu ammonis; DG - dentate gyrus; SRLM - stratum radiatum lacunosum moleculare.

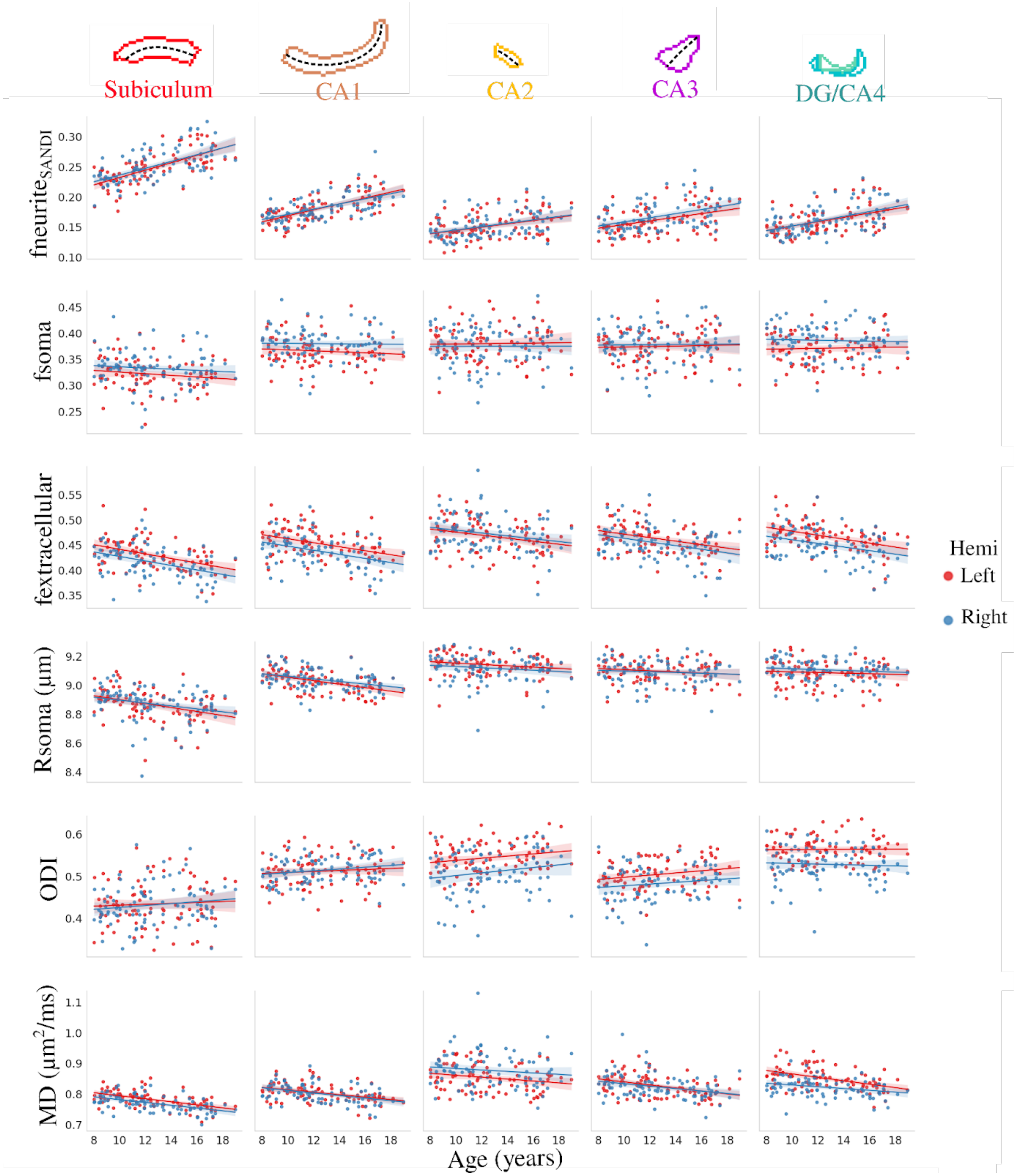
Subiculum
CA1
CA2
CA3
DG/CA4
$fneurite_{SANDI}$
fsoma
fextracellular
Rsoma (μm)
ODI
MD (μm^2/ms)
Age (years)
Hemi
Left
Right

Supplementary Figure S5. Relationship between age and subfield averaged microstructural measures of neurite ($fneurite_{SANDI}$), soma (fsoma), and extracellular (fextracellular) signal fractions, soma radius, orientation dispersion index (ODI) and mean diffusivity (MD) between the left and right hemisphere. The dashed lines approximately represent the midthickness surface which the metrics were sampled and then averaged on. CA - cornu ammonis; DG - dentate gyrus.

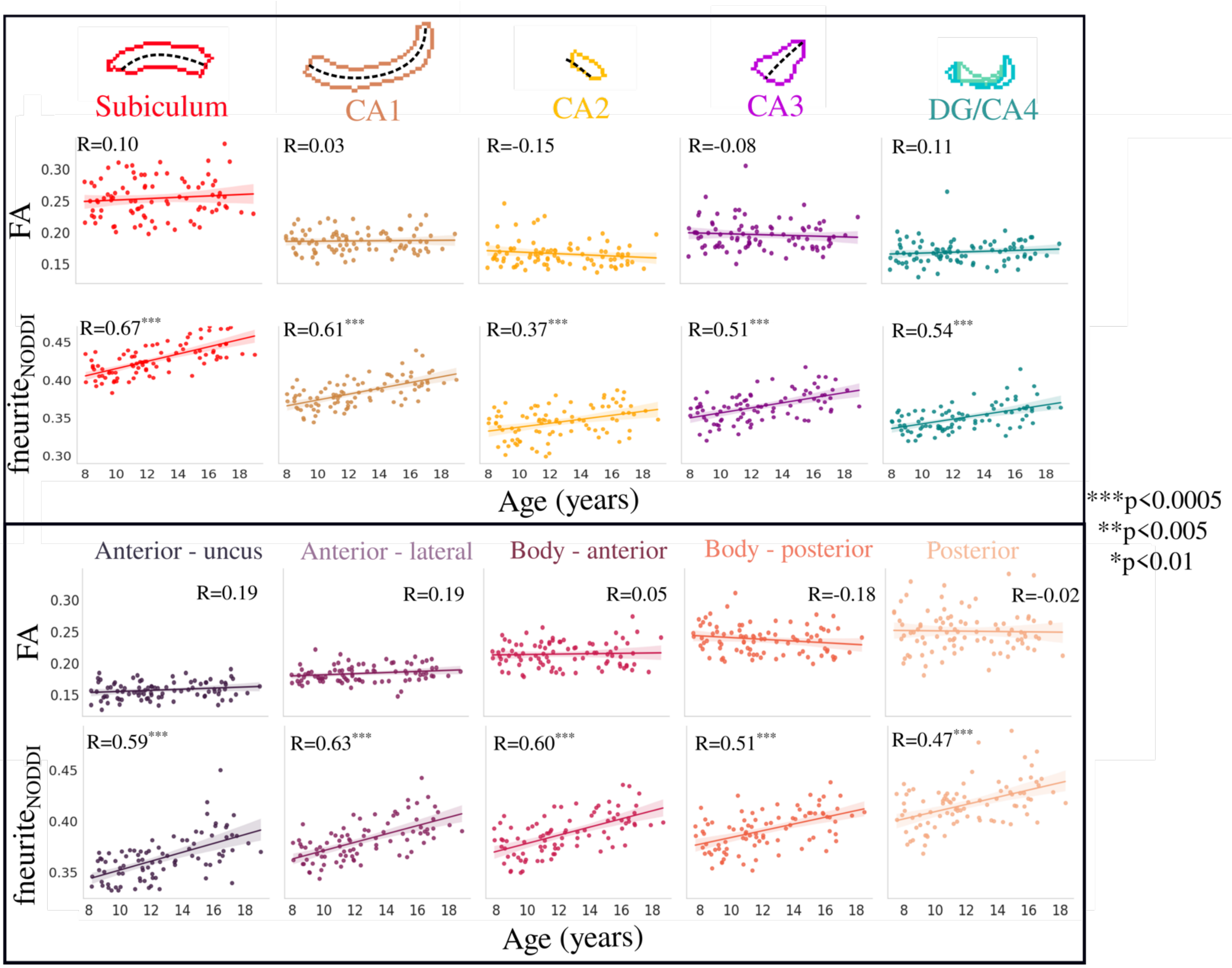

Supplementary Figure S6. Correlation between age and subfield (top) and long-axis (bottom) averaged microstructural measures of neurite signal fraction from NODDI ($neurite_{NODDI}$) and fractional anisotropy (FA). Relationships are quantified using Pearson's correlation coefficient (R) with the significance represented by asterisk(s) which is the probability that two uncorrelated variables could produce a correlation similar to the observed correlation value. The dashed lines approximately represent the midthickness surface which the metrics were sampled and then averaged on. CA - cornu ammonis; DG - dentate gyrus

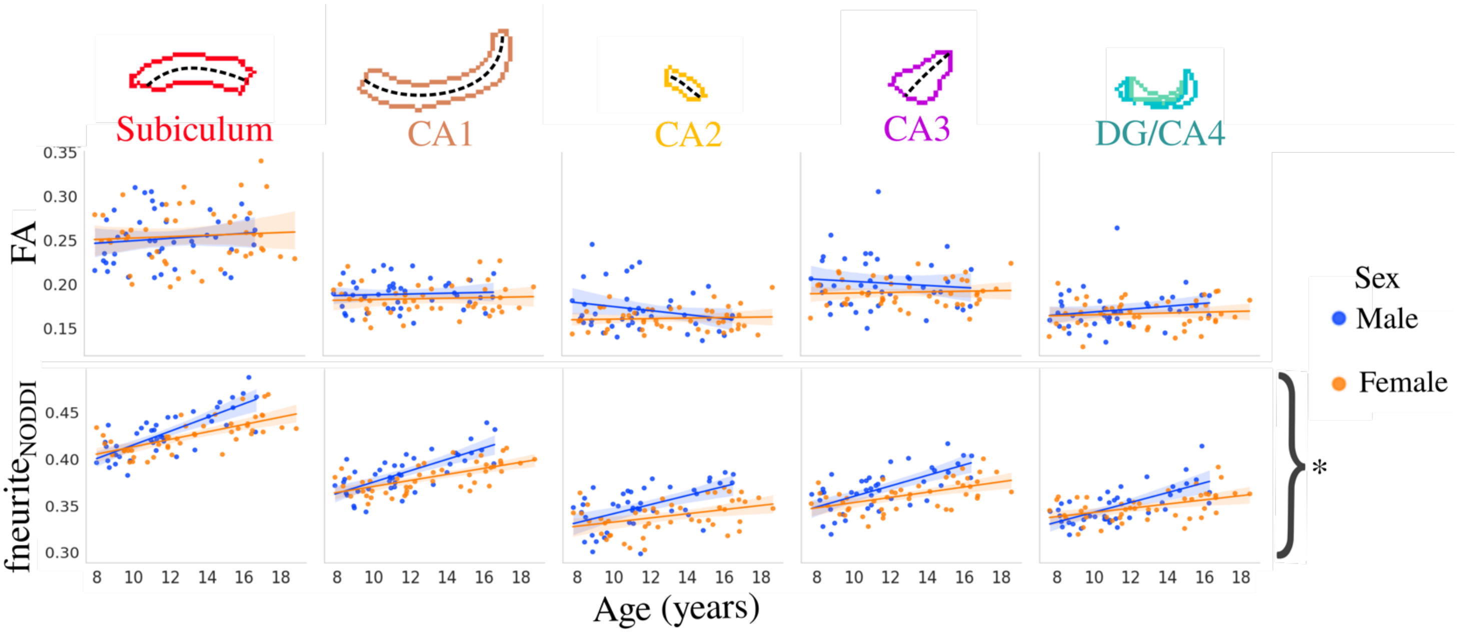

Supplementary figure S7. Relationship between age and microstructure by hippocampal subfield and stratified by sex. Though the subfields are plotted separately here, the asterisks represent metrics with a significant interaction between age and sex (i.e. subfields are combined) after FDR correction ($p<0.05$).

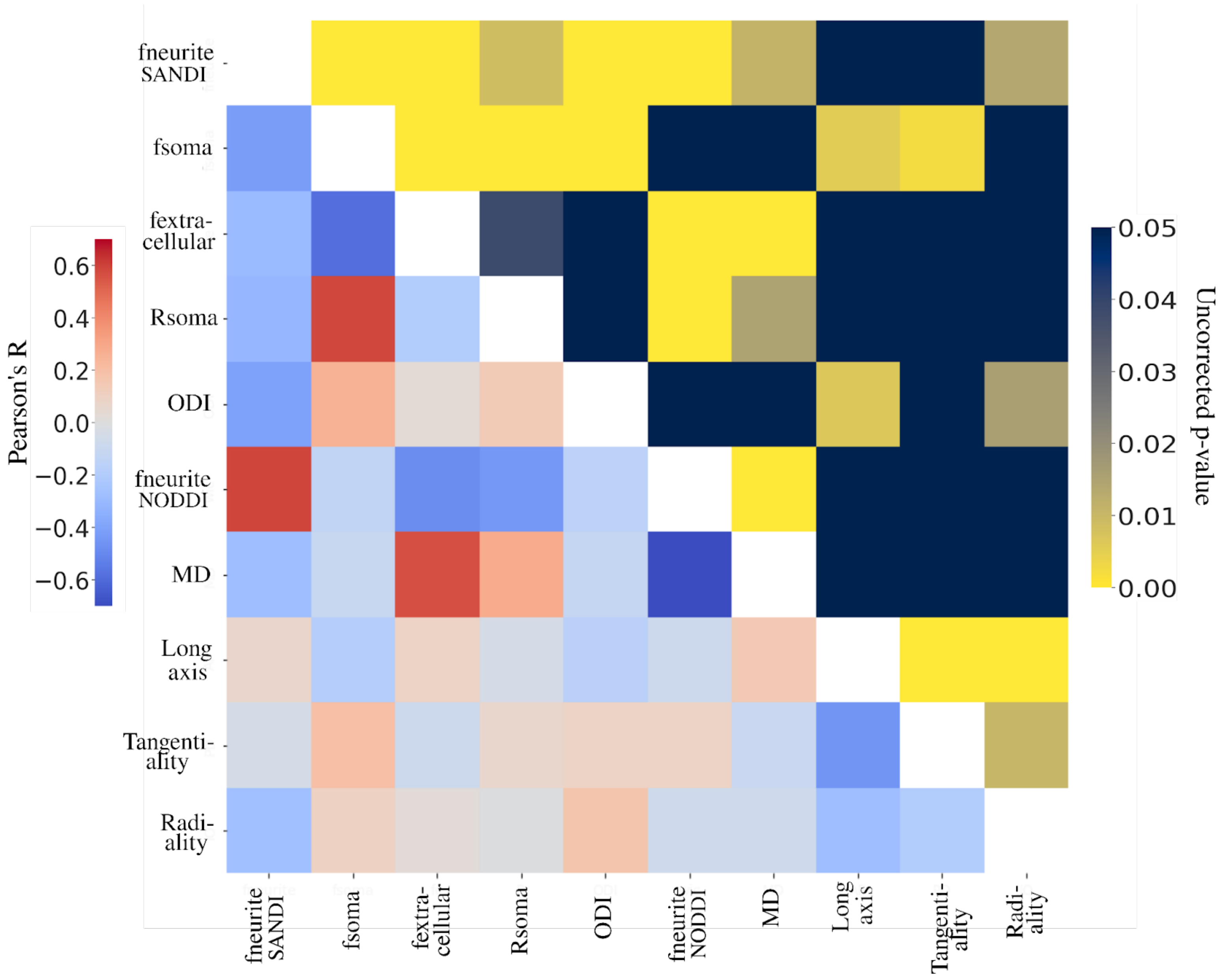

Supplementary Figure S8. Spin test correlation between all the age contrasted t-statistic microstructure maps which capture vertex-wise age-related microstructural changes.

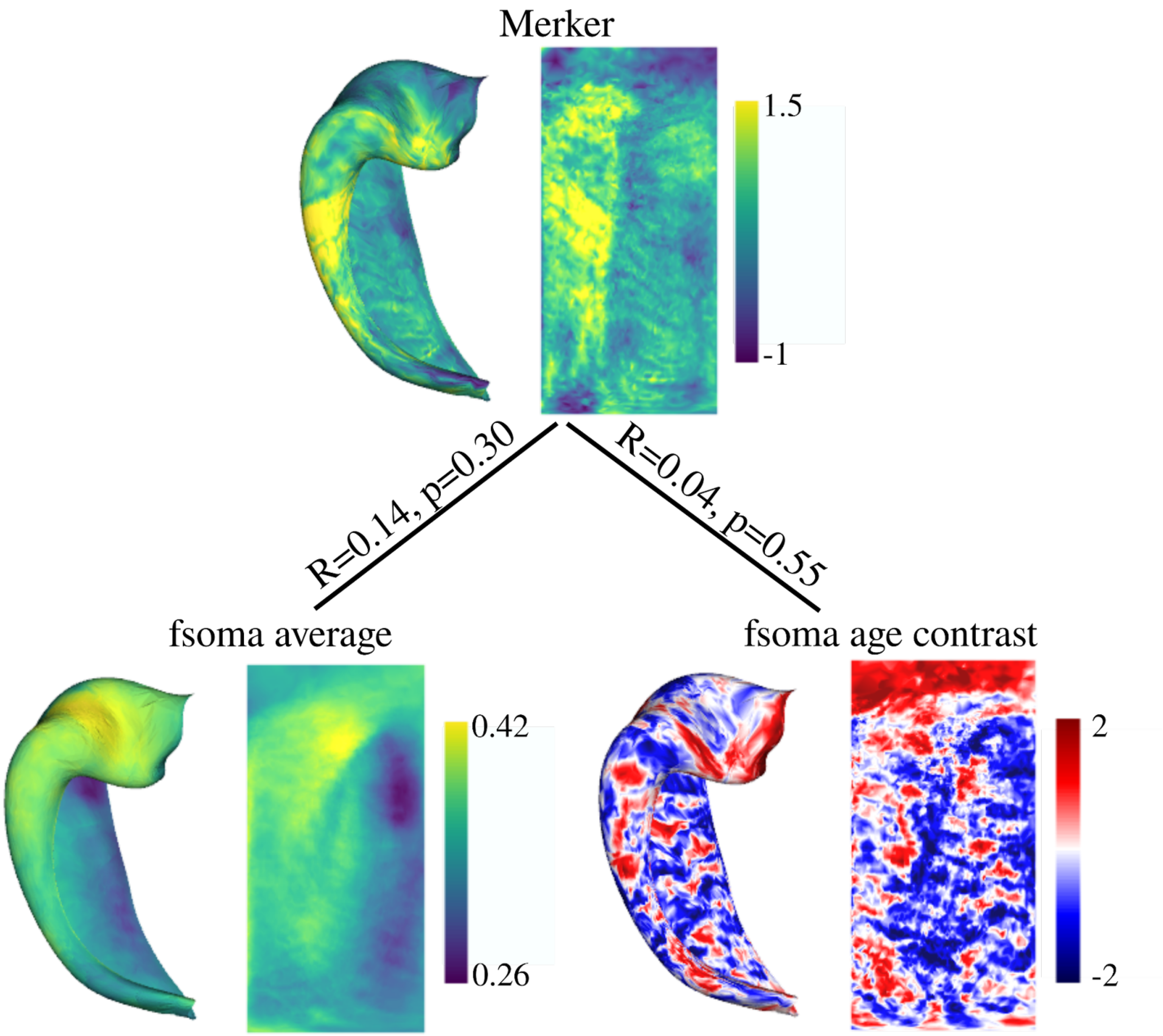

Supplementary Figure S9. Correlating a Merker stain for cell bodies with fsoma derived from SANDI (DeKraker et al., 2024; Amunts et al., 2013). Bottom left is fsoma averaged across all subjects (i.e. averaging across age) and bottom right is the age contrasted t-statistic map capturing vertex-wise age-related changes of fsoma.

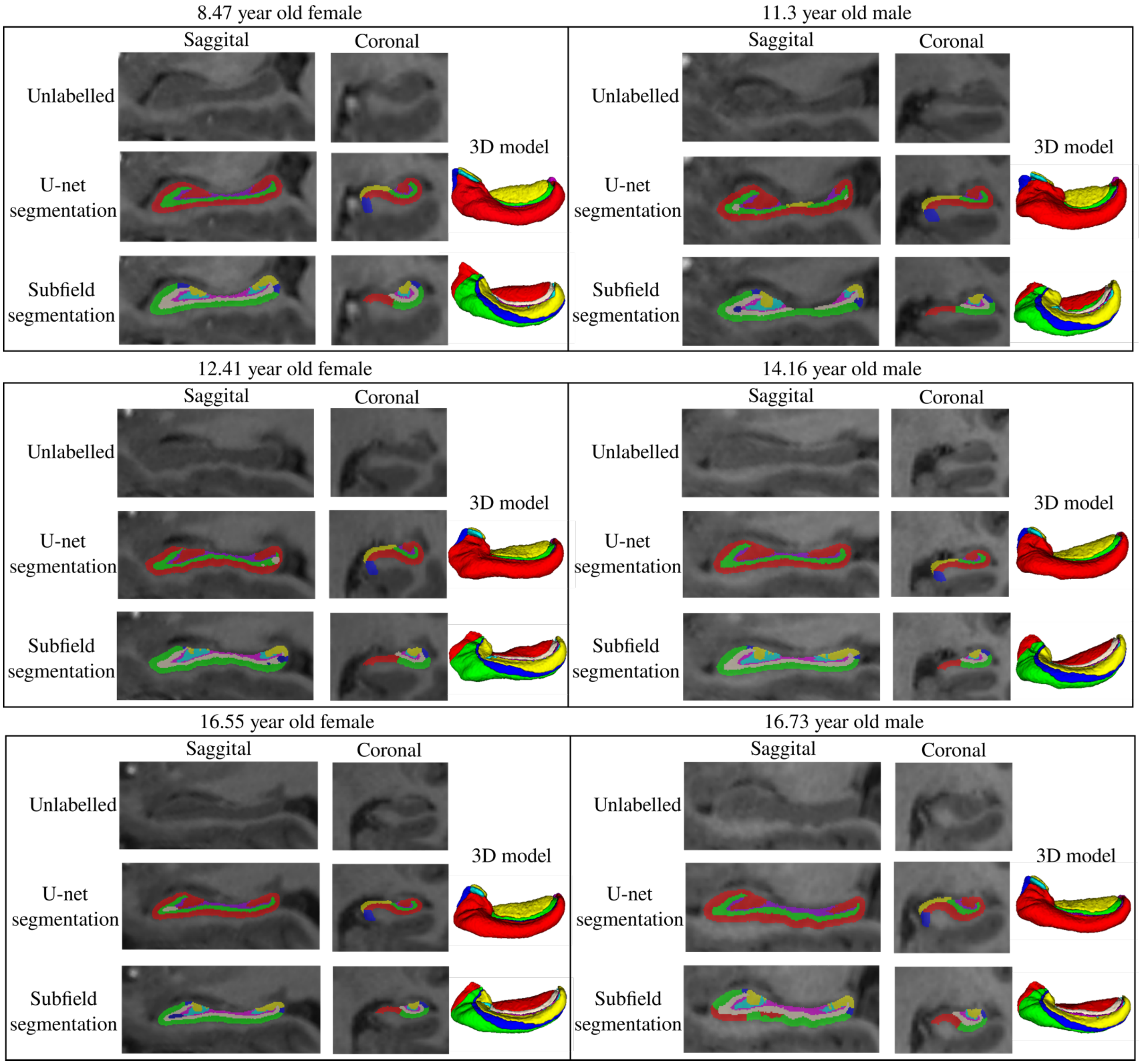

Supplementary Figure S10. Example cases of successful U-net and subsequent subfield segmentations from HippUnfold across varying age and sex. The colour labelling for the U-net segmentations is as follows: red - hippocampal gray matter, blue – medial temporal lobe cortex, yellow – pial surface, light blue – hippocampal-amygdala transition area, pink – indusium

griseum, green – SRLM, purple – dentate gyrus, white – cyst. The colour labelling for the subfields is as follows: red – subiculum, green – CA1, blue – CA2, yellow – CA3, light blue – CA4, pink – dentate gyrus, white – SRLM.

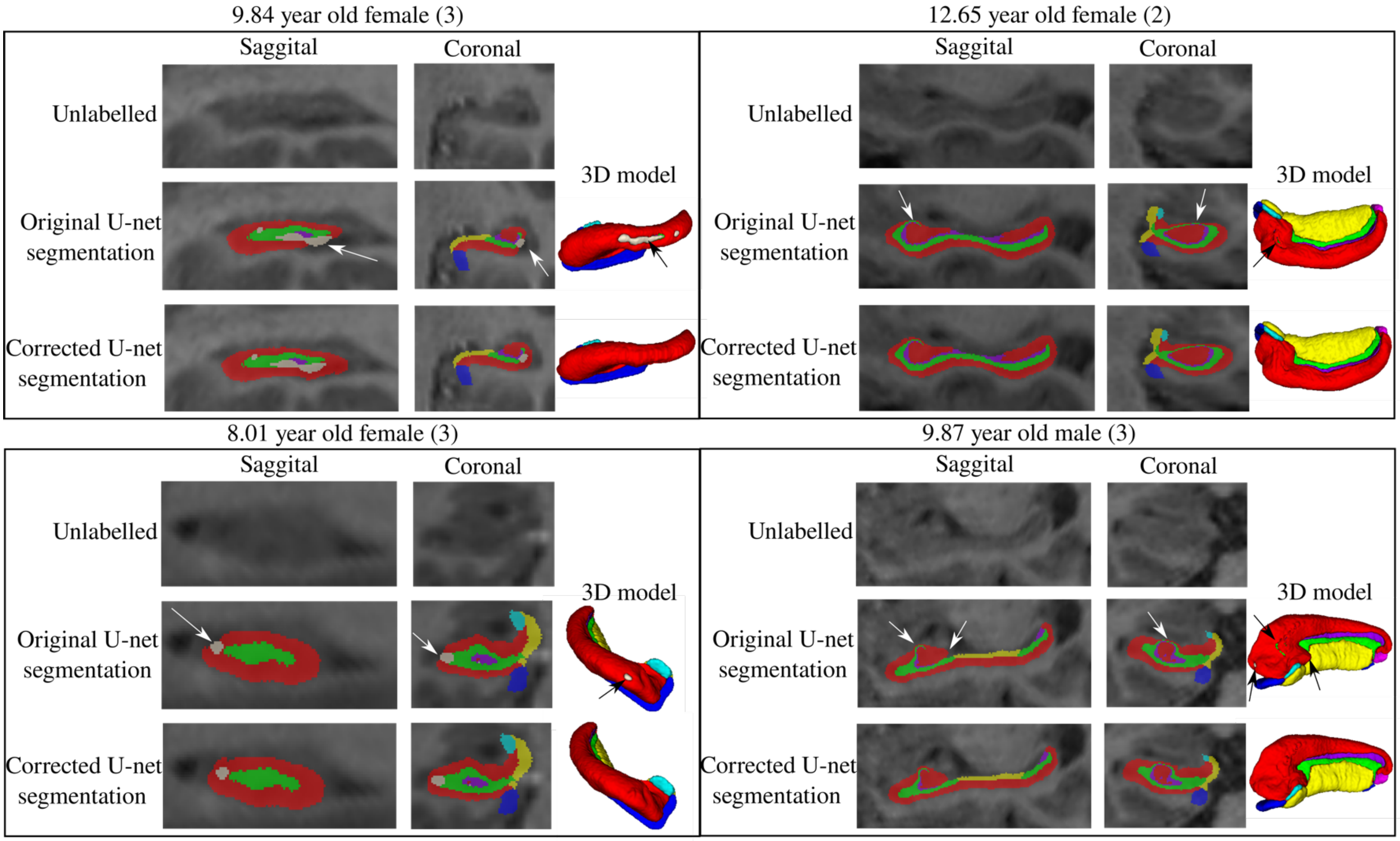

Supplementary Figure S11. Example cases of manually corrected U-net segmentations from HippUnfold. The colour labelling for the U-net segmentations is as follows: red - hippocampal gray matter, blue – medial temporal lobe cortex, yellow – pial surface, light blue – hippocampalamygdala transition area, pink – indusium griseum, green – SRLM, purple – dentate gyrus, white

– cyst. All segmentations were ranked on a scale of 1-3, with 1 being a high-quality segmentation requiring no manual correction and 3 being a decent quality segmentation that could use some manual correction. The integer in the brackets is the quality control score given to the original U-net segmentation. The black arrows correspond to the regions where manual correction was made.

Supplementary Table S1. Summary statistics for an F-test performed between a full and reduced linear model to examine the interaction between age and hemisphere. The form of the reduced model was: $metric_{reduced}$ = age + subfield + hemi + age:subfield + hemi:subfield and for the full model was: $metric_{full}$ = age + subfield + hemi + age:subfield + hemi:subfield + age:hemi.

Metric	Residual degrees of freedom	Difference in number of parameters between full and reduced models	F-value	Uncorrected p-value
Volume	864	1	0.001	0.974
Gyrification	864	1	0.104	0.747
Thickness	864	1	0.554	0.457
$fneurite_{SANDI}$	864	1	0.014	0.906
fsoma	864	1	0.001	0.971
fextracellular	864	1	0.014	0.907
Rsoma	864	1	0.576	0.448
ODI	864	1	0.089	0.766
MD	864	1	1.223	0.269

Abbreviations: $fneurite_{SANDI}$: neurite fraction from SANDI; fsoma: soma signal fraction; fextracellular: extracellular signal fraction; Rsoma: soma radius; ODI: orientation dispersion index from NODDI; MD: mean diffusivity from DTI.

Supplementary Table S2. Summary statistics for an F-test performed between a full and reduced linear model to examine the interaction between age and sex. The form of the reduced model was: $metric_{reduced}$ = age + subfield + sex + age:subfield + sex:subfield and for the full model was: $metric_{full}$ = age + subfield + sex + age:subfield + sex:subfield + age:sex.

Metric	Residual degrees of freedom	Difference in number of parameters between full and reduced models	F-value	FDR-corrected p-value
Volume	424	1	6.030	**0.040**
Gyrification	424	1	1.573	0.211
Thickness	424	1	4.966	**0.040**
$fneurite_{SANDI}$	424	1	43.39	**$8x10^{-10}$**
fsoma	424	1	2.673	0.103
fextracellular	424	1	4.509	**0.040**

Rsoma	424	1	16.819	**1.47×10^{-4}**
ODI	424	1	10.392	**0.002**
MD	424	1	14.247	**3.66×10^{-4}**

Bold p-values indicate $p < 0.05$ after false-discovery rate (FDR) correction. Abbreviations: $fneurite_{SANDI}$: neurite fraction from SANDI; fsoma: soma signal fraction; fextracellular: extracellular signal fraction; Rsoma: soma radius; ODI: orientation dispersion index from NODDI; MD: mean diffusivity from DTI.

Supplementary Table S3. Summary statistics for an F-test performed between a full and reduced linear model to examine the interaction between age and subfield. The form of the reduced model was: $metric_{reduced}$ = age + subfield + sex + age:sex + sex:subfield and for the full model was: $metric_{full}$ = age + subfield + sex + age:sex + sex:subfield + age:subfield.

Metric	Residual degrees of freedom	Difference in number of parameters between full and reduced models	F-value	FDR-corrected p-value
Volume	424	4	1.105	0.948
Gyrification	424	4	0.397	0.948
Thickness	424	4	0.181	0.948
$fneurite_{SANDI}$	424	4	4.35	**0.011**
fsoma	424	4	0.511	0.894
fextracellular	424	4	0.274	0.894
Rsoma	424	4	3.09	**0.048**
ODI	424	4	0.838	0.894
MD	424	4	0.281	0.894
Long-axis oriented diffusion	424	4	1.6	0.516
Tangential oriented diffusion	424	4	1.125	0.516
Radial oriented diffusion	424	4	0.767	0.547

Bold p-values indicate $p < 0.05$ after false-discovery rate (FDR) correction. Abbreviations: $fneurite_{SANDI}$: neurite fraction from SANDI; fsoma: soma signal fraction; fextracellular: extracellular signal fraction; Rsoma: soma radius; ODI: orientation dispersion index from NODDI; MD: mean diffusivity from DTI.

Supplementary Table S4. Summary statistics for an F-test performed between a full and reduced linear model to examine the interaction between age and long-axis region for the microstructural measures. The form of the reduced model was: $metric_{reduced}$ = age + long-axis + sex + age:sex + sex:long-axis and for the full model was: $metric_{full}$ = age + long-axis + sex + age:sex + sex:long-axis + age:long-axis.

Metric	Residual degrees of freedom	Difference in number of parameters between full and reduced models	F-value	FDR-corrected p-value
$fneurite_{SANDI}$	424	4	0.323	0.883
fsoma	424	4	0.292	0.883
fextracellular	424	4	0.373	0.883
Rsoma	424	4	0.804	0.883
ODI	424	4	2.054	0.516
MD	424	4	1.126	0.883
Long-axis oriented diffusion	424	4	3.190	**0.020**
Tangential oriented diffusion	424	4	2.187	0.070
Radial oriented diffusion	424	4	5.341	**0.001**

Bold p-values indicate $p < 0.05$ after false-discovery rate (FDR) correction. Abbreviations: $fneurite_{SANDI}$: neurite fraction from SANDI; fsoma: soma signal fraction; fextracellular: extracellular signal fraction; Rsoma: soma radius; ODI: orientation dispersion index from NODDI; MD: mean diffusivity from DTI.
