# Supplementary material for: Microstructural variation of hippocampal substructures across childhood and adolescence quantified with high-gradient diffusion MRI
Source: Commun Biol. 2026 Feb 12;9:416. doi: 10.1038/s42003-026-09622-x (PMC13009190; doi:10.1038/s42003-026-09622-x)
Supplement: Supplementary file 2 — Description of Additional Supplementary Files [file 42003_2026_9622_MOESM2_ESM.pdf]

Description of Additional Supplementary File

File name: Supplementary data 1

Description: A supplementary data excel file has been uploaded with the manuscript, containing numerical source data to reproduce each figure.
