# Supplementary material for: Microstructural variation of hippocampal substructures across childhood and adolescence quantified with high-gradient diffusion MRI
Source: Commun Biol. 2026 Feb 12;9:416. doi: 10.1038/s42003-026-09622-x (PMC13009190; doi:10.1038/s42003-026-09622-x)
Supplement: Supplementary file 4 — Reporting Summary [file 42003_2026_9622_MOESM4_ESM.pdf]

nature portfolio

Corresponding author(s): Bradley Karat

Last updated by author(s): Jan 11, 2026

Reporting Summary

Nature Portfolio wishes to improve the reproducibility of the work that we publish. This form provides structure for consistency and transparency in reporting. For further information on Nature Portfolio policies, see our Editorial Policies and the Editorial Policy Checklist.

Statistics

For all statistical analyses, confirm that the following items are present in the figure legend, table legend, main text, or Methods section.

n/a	Confirmed	
☐	☒	The exact sample size (*n*) for each experimental group/condition, given as a discrete number and unit of measurement
☐	☒	A statement on whether measurements were taken from distinct samples or whether the same sample was measured repeatedly
☐	☒	The statistical test(s) used AND whether they are one- or two-sided<br>*Only common tests should be described solely by name; describe more complex techniques in the Methods section.*
☐	☒	A description of all covariates tested
☐	☒	A description of any assumptions or corrections, such as tests of normality and adjustment for multiple comparisons
☐	☒	A full description of the statistical parameters including central tendency (e.g. means) or other basic estimates (e.g. regression coefficient) AND variation (e.g. standard deviation) or associated estimates of uncertainty (e.g. confidence intervals)
☐	☒	For null hypothesis testing, the test statistic (e.g. *F*, *t*, *r*) with confidence intervals, effect sizes, degrees of freedom and *P* value noted<br>*Give P values as exact values whenever suitable.*
☒	☐	For Bayesian analysis, information on the choice of priors and Markov chain Monte Carlo settings
☒	☐	For hierarchical and complex designs, identification of the appropriate level for tests and full reporting of outcomes
☐	☒	Estimates of effect sizes (e.g. Cohen's *d*, Pearson's *r*), indicating how they were calculated

Our web collection on statistics for biologists contains articles on many of the points above.

Software and code

Policy information about availability of computer code

Data collection	N/A
Data analysis	SANDI matlab toolbox (published latest version) to fit the SANDI model. HippUnfold (published; 1.2.0) to model the hippocampus. Used FSL (6.0.5) to fit the DTI model (published). Used the Microstructure Diffusion Toolbox (MDT; 1.2.7) to fit the NODDI model (published). FreeSurfer for volume analysis (7.2.0). MRtrix3 (3.0.3). Code to repeat the study analyses can be found at: https://github.com/Bradley-Karat/Developing_hippocampus.

For manuscripts utilizing custom algorithms or software that are central to the research but not yet described in published literature, software must be made available to editors and reviewers. We strongly encourage code deposition in a community repository (e.g. GitHub). See the Nature Portfolio guidelines for submitting code & software for further information.

Data

Policy information about availability of data

All manuscripts must include a data availability statement. This statement should provide the following information, where applicable:

- Accession codes, unique identifiers, or web links for publicly available datasets
- A description of any restrictions on data availability
- For clinical datasets or third party data, please ensure that the statement adheres to our policy

Due to the inclusion of minors (under 18 participants), the availability of derived or identifiable data from the participant cohort is restricted due to privacy

concerns. Derived data supporting the findings of the imaging analyses are available by contacting the authors in writing via email. Source data for reproducing all figures are provided with this paper.

Research involving human participants, their data, or biological material

Policy information about studies with human participants or human data. See also policy information about sex, gender (identity/presentation), and sexual orientation and race, ethnicity and racism.

Reporting on sex and gender	This study has analyses stratified by biological sex which was given by parental reporting.
Reporting on race, ethnicity, or other socially relevant groupings	No race, ethnicity or other socially relevant groupings was used.
Population characteristics	Age and sex of the healthy children and adolescents was obtained.
Recruitment	The children were recruited as part of the Cardiff University Brain Research Imaging Centre (CUBRIC) Kids study.
Ethics oversight	The study was approved by the School of Psychology ethics committee at Cardiff University.

Note that full information on the approval of the study protocol must also be provided in the manuscript.

Field-specific reporting

Please select the one below that is the best fit for your research. If you are not sure, read the appropriate sections before making your selection.

☒ Life sciences ☐ Behavioural & social sciences ☐ Ecological, evolutionary & environmental sciences

For a reference copy of the document with all sections, see nature.com/documents/nr-reporting-summary-flat.pdf

Life sciences study design

All studies must disclose on these points even when the disclosure is negative.

Sample size	The sample size of 88 was partly chosen based on resources, recruitment, time, and comparison to studies of similar analyses.
Data exclusions	Children were excluded from the study if they had repeated history of major head injuries or epilepsy (as this could result in brain changes unrelated to aging), or if they had any contraindication to MRI (i.e. metal implants).
Replication	Analyses of the same dataset were performed which compared diffusion metrics to simulated data (Genc et al., 2020) which revealed correspondence between derived metrics and simulations. Six healthy adults aged 24-30 years (3 female) were scanned five times in the span of two weeks (Koller et al., 2021) on the same MRI system, where repeatability of the metrics used in this study were seen.
Randomization	Participants and their parents/guardians were recruited via public outreach events, and there is no group assignment or intervention in this study.
Blinding	The analysis pipeline was the same for all participants, and the investigators running the analysis did not analyze the age of each subject until after all processing was complete.

Reporting for specific materials, systems and methods

We require information from authors about some types of materials, experimental systems and methods used in many studies. Here, indicate whether each material, system or method listed is relevant to your study. If you are not sure if a list item applies to your research, read the appropriate section before selecting a response.

Materials & experimental systems

n/a	Involved in the study	
☒	☐	Antibodies
☒	☐	Eukaryotic cell lines
☒	☐	Palaeontology and archaeology
☒	☐	Animals and other organisms
☒	☐	Clinical data
☒	☐	Dual use research of concern
☒	☐	Plants

Methods

n/a	Involved in the study	
☒	☐	ChIP-seq
☒	☐	Flow cytometry
☐	☒	MRI-based neuroimaging

Plants

Seed stocks	Plants not included.
Novel plant genotypes	N/A
Authentication	N/A

Magnetic resonance imaging

Experimental design

Design type	Cross-sectional data with no task performed.
Design specifications	Each participant was scanned once with a diffusion and structural protocol.
Behavioral performance measures	N/A

Acquisition

Imaging type(s)	Structural, diffusion.
Field strength	3T
Sequence & imaging parameters	Structural T1-weighted at voxel-size=1x1x1mm3; TE/TR=2/2300 ms. Multi-shell dMRI (TE/TR=59/3000 ms; voxel-size=2x2x2 mm3; Δ = 23.3 ms, δ = 7 ms, b-values = 0 (14 vols), 500, 1200(30 dirs), 2400, 4000, 6000 (60 dirs) s/mm2).
Area of acquisition	Whole-brain.

Diffusion MRI ☒ Used ☐ Not used

Parameters: Multi-shell dMRI (TE/TR=59/3000 ms; voxel-size=2x2x2 mm3; Δ = 23.3 ms, δ = 7 ms, b-values = 0 (14 vols), 500, 1200(30 dirs), 2400, 4000, 6000 (60 dirs) s/mm2) with no cardiac gating.

Preprocessing

Preprocessing software	Pre-processing of dMRI data involved steps largely in line with recommended steps for standard 3.0T systems, interfacing various tools such as FSL, MRtrix3, and ANTS. These steps included: denoising, slicewise outlier detection (SOLID), and correction for drift; motion, eddy, and susceptibility-induced distortions; Gibbs ringing artefact; bias field; and gradient nonlinearities. Root mean squared (RMS) displacement from eddy was used as a summary measure of global head motion. Estimates of SNR were performed by taking the signal in the white matter and dividing this by the signal outside of the brain (for each b =0 image). SNR estimates in the in vivo data were: mean = 48.02, SD =7.46.T1-weighted data were processed using FreeSurfer version 6.0
Normalization	Data was not standardized, as it was analyzed in each subjects native space with their own hippocampal representation since the HippUnfold software works in the subjects native space.
Normalization template	N/A
Noise and artifact removal	Denoising, slicewise outlier detection (SOLID), and correction for drift; motion, eddy, and susceptibility-induced distortions; Gibbs ringing artefact; bias field; and gradient nonlinearity correction was performed. Root mean squared (RMS) displacement from eddy was used as a summary measure of global head motion.
Volume censoring	N/A

Statistical modeling & inference

Model type and settings	Pearsons correlation coefficient and multivariate general linear modelling, and vertex-based statistics using a bespoke spin test previously published was used.
Effect(s) tested	The effect of age was tested.

Specify type of analysis: ☐ Whole brain ☒ ROI-based ☐ Both

Anatomical location(s): The hippocampus was analyzed using the published HippUnfold software.

Statistic type for inference (See Eklund et al. 2016): Subfield-based (ROI) and vertex-wise analyses were performed.

Correction: FDR correction was performed.

Models & analysis

n/a	Involved in the study
☒	☐ Functional and/or effective connectivity
☒	☐ Graph analysis
☐	☒ Multivariate modeling or predictive analysis

Multivariate modeling and predictive analysis: Multivariate models were built to assess the change in microstructure as a function of age, subfield, and sex.
